# Pharmacologically inherited carbon dots from *Salvia miltiorrhiza* with potent antioxidant activity and multi-pathway modulation for myocardial ischemia-reperfusion injury therapy

**DOI:** 10.7150/thno.123141

**Published:** 2026-01-01

**Authors:** Kai Zhang, Zhenyuan Wang, Letong Zhang, Hao Wu, Jing Liu, Mingzhen Zhang, Zhichao Deng, Ruina Liu

**Affiliations:** 1 NHC Key Laboratory of Forensic Science, Department of Forensic Pathology, College of Forensic Medicine, Xi'an Jiaotong University, 76 Yanta West Road, Xi'an, 710061, People's Republic of China.; 2 Center for Translational Medicine, Shaanxi Provincial Key Laboratory of Biological Psychiatry, Department of Psychiatry, The First Affiliated Hospital of Xi'an Jiaotong University, 277 Yanta West Road, Xi'an, 710061, People's Republic of China.; 3 School of Basic Medical Sciences, Xi'an Jiaotong University, 76 Yanta West Road, Xi'an, 710061, People's Republic of China.

**Keywords:** carbon dots, nanozymes, chinese herbal medicine, myocardial ischemia-reperfusion injury, oxidative stress

## Abstract

**Rationale:** Myocardial ischemia-reperfusion (I/R) injury remains a major clinical challenge that limits the efficacy of reperfusion therapy in acute myocardial infarction, mainly due to excessive production of reactive oxygen species (ROS) and the resulting oxidative stress, inflammation, and cardiomyocyte death. However, conventional antioxidant strategies show limited clinical efficacy, highlighting the urgent need for novel redox-regulating therapies.

**Methods:** We synthesized carbon dot nanozymes (SM-CDs) via a green hydrothermal process using *Salvia miltiorrhiza*, a traditional Chinese medicinal herb. Their size, structure, and antioxidant enzymatic activities were thoroughly characterized. The contribution of surface functional groups to the superoxide dismutase (SOD)-like activity of SM-CDs were investigated by surface modification. *In vitro* antioxidant, anti-inflammatory, and anti-apoptotic effects were evaluated in RAW264.7 macrophages and H9C2 cardiomyocytes. *In vivo* therapeutic effects were accessed in a rat myocardial I/R model. Transcriptomics analysis was used to explore underlying cardioprotective mechanisms. Network pharmacology analysis was employed to study potential pharmacological activity inherited from the herbal precursor.

**Results:** SM-CDs exhibit potent ROS-scavenging capacity, with surface carbonyl and hydroxyl groups playing key roles in their remarkable SOD-like activity. *In vitro*, SM-CDs effectively scavenged intracellular ROS, suppressed macrophage M1 polarization, and attenuated cardiomyocyte apoptosis. *In vivo*, intramyocardial injection of SM-CDs significantly reduced inflammation, apoptosis, and infarct size, while improving cardiac remodeling and functional recovery through fibrosis inhibition and enhanced neovascularization. These effects were potentially associated with inhibition of NF-κB and NOD-like receptor signaling pathways and activation of PI3K-Akt and FoxO pathways. Strong pathway concordance between SM-CD-regulated pathways and known therapeutic targets of *Salvia miltiorrhiza* suggests that SM-CDs may retain pharmacological activity from their herbal precursor.

**Conclusions:** This study introduces SM-CDs as biocompatible nanozymes with potent antioxidant and cardioprotective potential for myocardial I/R injury.

## Introduction

Myocardial infarction (MI), the most severe clinical manifestation of coronary artery disease, remains a leading cause of mortality worldwide despite a gradual decline in incidence over recent decades 1, 2. Epidemiological data indicate a persistent prevalence of 3.8% among individuals under 60 years of age and 9.5% among those above 60 3. Although reperfusion therapies such as percutaneous coronary intervention and thrombolysis have improved survival rates, their therapeutic benefits are often compromised by the paradoxical phenomenon of ischemia-reperfusion (I/R) injury, which exacerbates myocardial damage during the restoration of blood flow 4. A key driver of myocardial I/R injury is the excessive generation of reactive oxygen species (ROS), which induce mitochondrial dysfunction and disrupt the integrity of cellular membranes in cardiomyocytes, ultimately leading to apoptotic or necrotic cell death 5. Additionally, ROS amplify inflammatory cascades by activating NF-κB signaling, thereby promoting the release of pro-inflammatory cytokines from macrophages 6. Elevated ROS levels following reperfusion trigger a detrimental cycle of oxidative stress, mitochondrial impairment, inflammation, and cell death, collectively aggravating myocardial injury. However, current antioxidant therapies have shown limited clinical efficacy 7. This underscores an urgent need for novel strategies capable of efficiently scavenging ROS, restoring redox homeostasis, and ultimately mitigating I/R-induced cardiac damage.

Nanozymes are a class of nanoscale materials that exhibit enzyme-mimicking catalytic activities. They have emerged as promising alternatives to natural enzymes due to their facile synthesis, excellent stability, tunable catalytic properties, and cost-effectiveness 8, 9. Among various catalytic types, redox nanozymes have attracted particular attention. Peroxidase (POD)-like nanozymes are widely explored for antibacterial and anticancer therapies by generating ROS to induce oxidative damage in cancer cells or pathogens 10-12, whereas catalase (CAT)- and superoxide dismutase (SOD)-like nanozymes are mainly applied for antioxidative purposes 13. Currently, various types of antioxidant nanozymes have been developed, including inorganic-based, MOF-based, and organic-based nanozymes. Among these, carbon dots (CDs) represent a subclass of carbon-based nanozymes with typical sizes below 10 nm. Compared with other types of nanozymes, CDs have several advantages, including superior catalytic performance, modifiable surface chemistry, excellent water solubility, and high biocompatibility 14. Certain types of CDs exhibit SOD-like activity, enabling efficient scavenging of superoxide anion radicals (•O_2_^-^), a major type of ROS. Studies have demonstrated that SOD-mimetic CDs effectively alleviate oxidative stress and cellular injury by neutralizing ROS, highlighting their therapeutic potential for treating oxidative stress-related diseases 15-18. Although other types of nanozymes, such as single-atom nanozymes, metal organic framework-based nanozymes, and Prussian blue nanozymes have shown therapeutic promise in mitigating myocardial I/R injury 19-22, investigations into the protective effects of CDs in this context are limited.

CDs synthesis strategies are broadly categorized into two types: top-down methods (e.g., laser ablation and electrochemical oxidation), which breakdown bulk carbon materials into small CDs, and bottom-up approaches (e.g., hydrothermal, solvothermal, or microwave-assisted carbonization), which utilize small organic molecules or oligomers as carbon precursors 23. However, these methods often involve strong oxidizing agents, toxic reagents, or organic solvents, posing environmental and safety concerns 24. In response, researchers have turned to greener synthesis approaches, using low-toxicity, heteroatom-rich natural biomass as carbon precursors. Traditional Chinese herbal medicines are considered ideal candidates for bottom-up synthesis because of their abundance, biocompatibility, low toxicity, cost-efficiency, and intrinsic pharmacological properties 25, 26. CDs derived from such herbal sources have diverse bioactivities, including antibacterial, hemostatic, antitumor, antioxidant, and anti-inflammatory effects 27-29, as well as protective effects in various types of I/R injury 30-34. Importantly, these bioactivities may be partially attributed to the inherent medicinal properties of the herbal precursors. *Salvia miltiorrhiza*, a widely used traditional Chinese herb for treating cardiovascular and cerebrovascular diseases, contains bioactive constituents primarily classified as tanshinones and salvianolic acids, which possess anti-inflammatory and anti-apoptotic properties 35. These compounds have demonstrated cardioprotective effects against myocardial infarction and I/R injury by modulating signaling pathways such as the PI3K/Akt, ​Akt/Erk1/2/Nrf2, and ​JAK2/STAT3 pathways 36. Therefore, synthesizing CDs using *Salvia miltiorrhiza* as a carbon precursor offers a highly promising strategy for the treatment of myocardial I/R injury.

**Figure 1:**
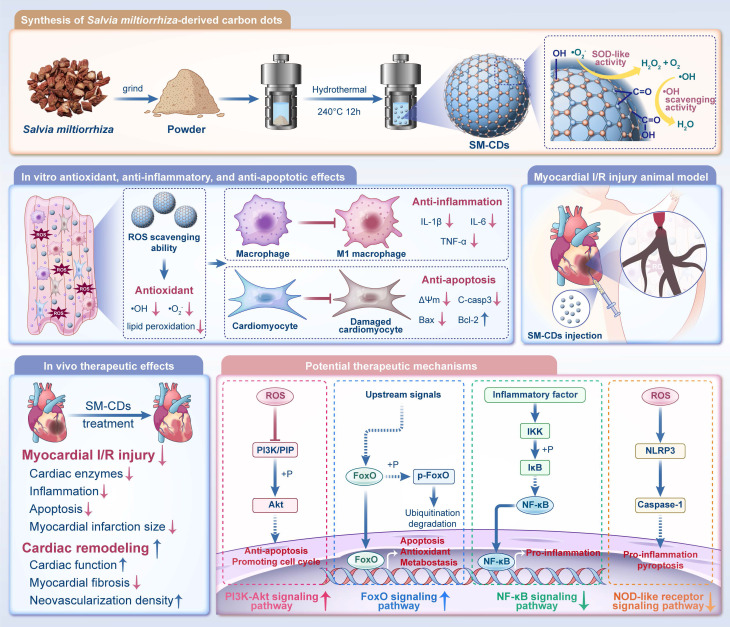
**Schematic illustration of the synthesis of SM-CDs and their therapeutic application in myocardial I/R injury.** SM-CDs were synthesized via a green hydrothermal method using *Salvia miltiorrhiza* as a natural carbon precursor. The resulting ultrasmall SM-CDs exhibited exceptional SOD-like activity, to which surface carbonyl and hydroxyl groups made significant contributions. SM-CDs display potent antioxidant properties and suppress pro-inflammatory M1 macrophage polarization, thereby reducing the release of pro-inflammatory cytokines. In cardiomyocytes, SM-CDs exert both antioxidant and anti-apoptotic effects, effectively mitigating cellular injury. Upon intramyocardial injection in a rat model of myocardial I/R injury, SM-CDs significantly alleviated myocardial damage and promoted cardiac repair and remodeling, potentially through the inhibition of NF-κB and NOD-like receptor signaling pathways and the activation of PI3K-Akt and FoxO pathways.

In this study, we synthesized *Salvia miltiorrhiza*-derived CDs (SM-CDs) via a hydrothermal method (Figure 1). SM-CDs with ultrasmall particle sizes​ exhibited potent •OH scavenging capacity and ​exceptional SOD-like activity​ for •O_2_⁻ elimination. Surface modification experiments highlighted the essential involvement of carbonyl and hydroxyl groups in mediating the antioxidant catalytic properties of SM-CDs. SM-CDs demonstrated both ​antioxidant and anti-inflammatory effects​ in macrophages, as well as ​antioxidant and ​anti-apoptotic effects​ in cardiomyocytes. In a rat model of myocardial I/R injury, SM-CDs significantly reduced myocardial damage, improved cardiac remodeling, and promoted functional recovery. Transcriptomic analysis indicated that SM-CDs exert cardioprotective effects through multiple mechanisms, including the suppression of the NF-κB and NOD-like receptor signaling pathways and the activation of the PI3K-Akt and FoxO pathways. Notably, 89.6% of the KEGG pathways modulated by SM-CDs overlapped with the predicted therapeutic targets of *Salvia miltiorrhiza* against myocardial I/R injury, as identified via network pharmacology analysis, suggesting that SM-CDs preserve key pharmacological attributes of their herbal precursor. Furthermore, the SM-CDs exhibited excellent biocompatibility in both *in vitro* and *in vivo* evaluations. Collectively, our findings present a green-synthesized, SOD-mimetic nanozyme derived from *Salvia miltiorrhiza* as a novel and effective therapeutic strategy for managing myocardial I/R injury.

## Results and Discussion

### Characterization of SM-CDs

**Figure 2:**
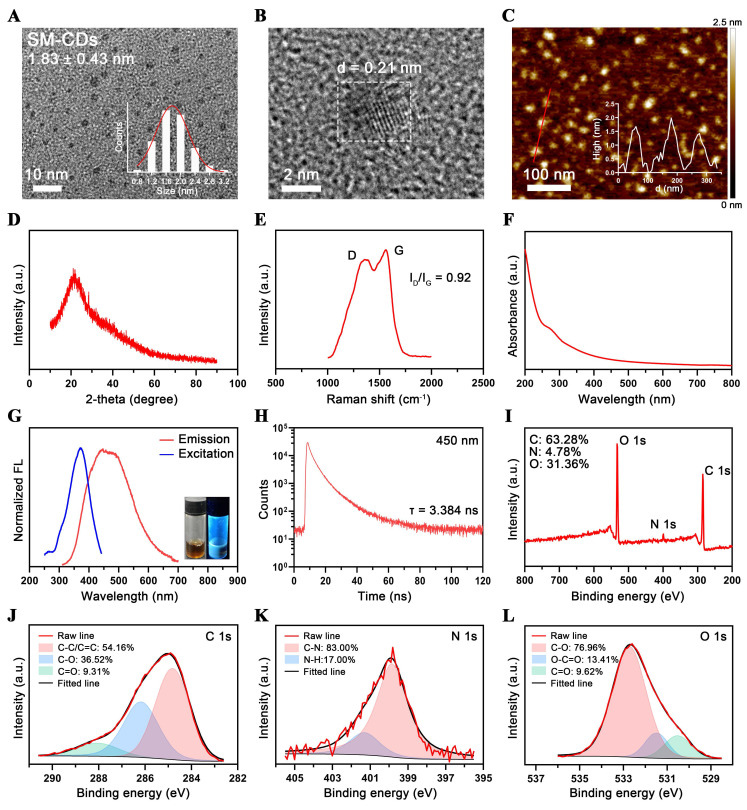
** Characterization of SM-CDs.** (A) TEM image and corresponding particle size distribution histogram of SM-CDs. (B) HR-TEM image showing lattice spacing of SM-CDs. (C) AFM image and corresponding height distribution of SM-CDs. (D) XRD spectrum of SM-CDs. (E) Raman spectrum of SM-CDs. (F) UV-vis absorption spectrum of SM-CDs. (G) Excitation and Emission spectra of SM-CDs (inset: photograph of SM-CDs solution under UV light excitation). (H) Fluorescence decay curve of SM-CDs was recorded at an emission wavelength of 450 nm. (I) XPS survey spectra of SM-CDs. (J-L) HR XPS spectra of C 1s, N 1s, and O 1s with peak fitting.

In this study, SM-CDs were synthesized from the traditional Chinese medicine *Salvia miltiorrhiza* using a one-pot hydrothermal method. Transmission electron microscopy (TEM) analysis revealed that the SM-CDs are well-dispersed, quasi-spherical nanoparticles with an average diameter of 1.83 ± 0.43 nm (Figure 2A). The high-resolution TEM image of SM-CDs (Figure 2B) showed a lattice spacing of 0.21 nm, corresponding to the (100) facets of graphite, confirming their crystalline structure 37. Atomic force microscopy (AFM) results further demonstrated that the height of SM-CDs ranged from approximately 1.5 to 2 nm, consistent with their quasi-spherical morphology (Figure 2C). X-Ray diffraction (XRD) analysis revealed a broad (002) peak centered at 21.8° (Figure 2D), corresponding to an interlayer spacing of approximately 0.41 nm. This value is larger than that of crystalline graphite, which is likely due to structural disorder, oxygen-rich surface functionalization, and the low graphitization characteristic of carbon dots. Raman spectroscopy exhibited the G- and D-bands with I_D_/I_G_ of 0.92 (Figure 2E), indicating a large portion of structural defects on the surface of SM-CDs. Additionally, SM-CDs exhibited a hydrodynamic diameter of 6.27 nm (Figure S1). The zeta potential was measured to be -27.5 mV, and the polydispersity index (PDI) was 0.079, indicating their high colloidal stability.

The optical properties of SM-CDs were investigated. The UV-vis absorption spectrum (Figure 2F) demonstrated broad absorption in the UV-vis region, attributed to π-π* transition (from sp^2^ hybridization in the carbon core) and n-π* transition (from surface functional groups) in the CDs 38-40. As shown in Figure 2G, the excitation and emission spectra of SM-CDs revealed a broad emission peak centered around 450 nm under an optimal excitation wavelength of 370 nm. The fluorescence emission exhibited excitation-dependent behavior, with the emission peak shifting according to the excitation wavelength (Figure S2). The fluorescence decay curve recorded at 450 nm yielded an average lifetime of 3.384 ns (Figure 2H).

X-ray photoelectron spectroscopy (XPS) was employed for semi-quantitative analysis of the elemental composition and chemical structure of SM-CDs. As shown in Figure 2I, the XPS spectrum exhibited three major peaks at 285, 400, and 532 eV, corresponding to C 1s, N 1s, and O 1s, respectively, revealing a molar composition of 63.28% C, 4.78% N, and 31.36% O. High-resolution (HR) XPS spectra of the C 1s, N 1s, and O 1s regions were further deconvoluted and analyzed (Figure 2J-L). The HR-C 1s spectrum exhibited binding energy peaks at 284.8, 286.2, and 288.1 eV, assigned to C-C/C=C, C-O, and C=O bonds, respectively. The HR-N 1s spectrum contained two peaks at 399.9 and 401.4 eV, corresponding to C-N and N-H bonds, respectively. In the HR-O 1s spectrum, the binding energy peaks at 530.5, 531.5, and 532.7 eV were attributed to C=O, O-C=O, and C-O, respectively. The carbon-to-oxygen atomic ratio of SM-CDs was determined to be 2.02, indicating a relatively high carbon content and suggesting a high degree of graphitization. Combined with the dominant C-C/C=C bonding pattern, this implies the presence of a large π-electron system, which may facilitate efficient electron transfer and stabilize radical intermediates, thereby enhancing SOD-like enzymatic activity 41. Moreover, the nitrogen content was found to be very low, indicating a negligible presence of nitrogen-containing heterocycles and amino groups. These findings confirm that SM-CDs possess a graphitized carbon core enriched with surface oxygen-containing functional groups, primarily carbonyl, hydroxyl, and carboxyl groups, which are likely introduced through oxidative etching during synthesis.

### Antioxidant enzyme activity of SM-CDs

**Figure 3:**
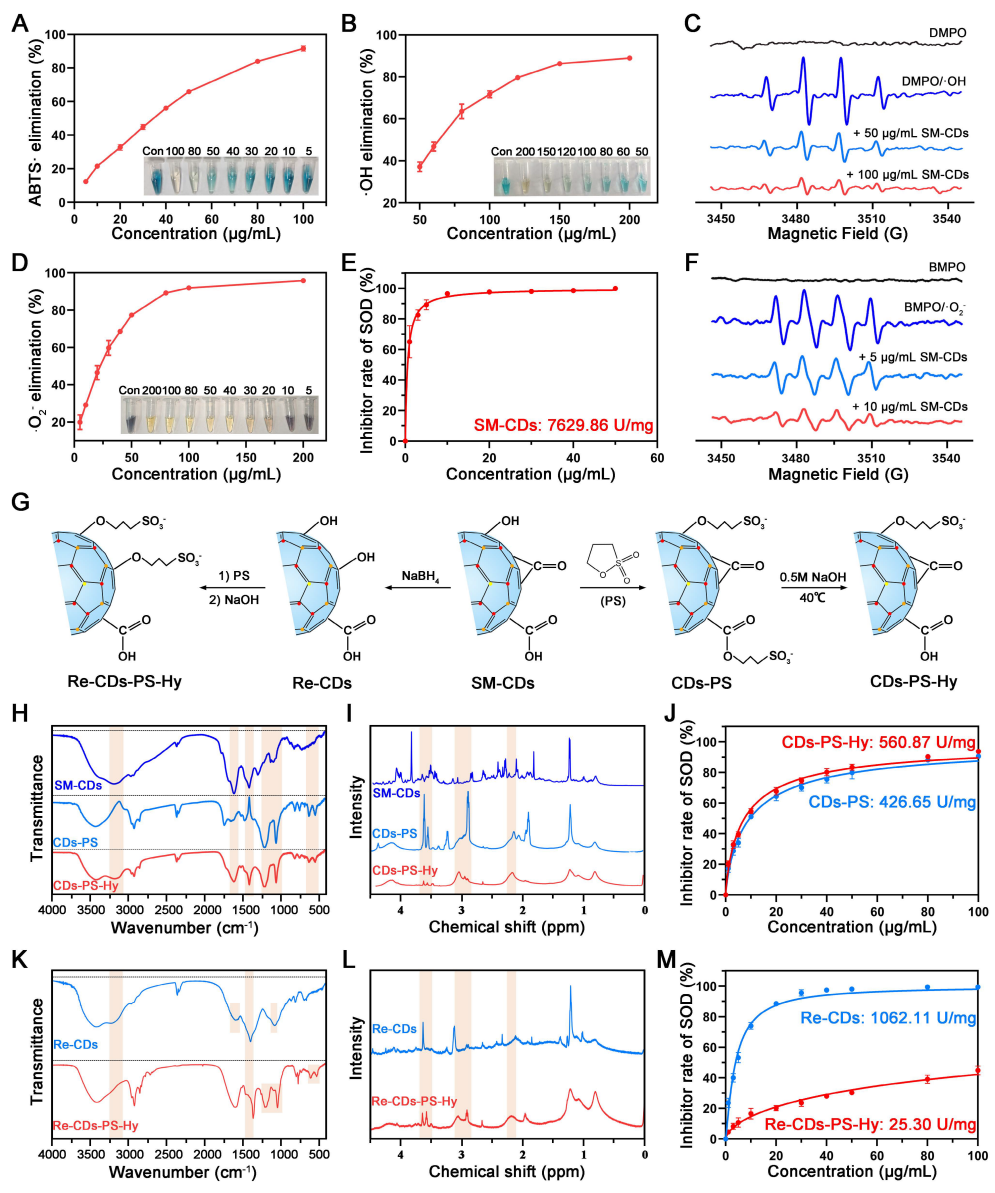
** ROS scavenging activity of SM-CDs and surface modifications for investigating structure‒activity relationships.** (A) Total antioxidant capacity of SM-CDs at concentrations ranging from 5 to 100 µg/mL (inset: concentration-dependent decolorization of ABTS•^+^). (B) •OH scavenging activity of SM-CDs at 50-200 µg/mL (inset: concentration-dependent decolorization of oxTMB). (C) EPR spectra showing •OH scavenging by SM-CDs at 50 and 100 μg/mL. (D) •O_2_^-^ scavenging activity of SM-CDs at 5-200 µg/mL (inset: concentration-dependent decolorization of formazan). (E) SOD-like activity of SM-CDs at physiological pH 7.4. (F) EPR spectra showing •O_2_^-^ scavenging by SM-CDs at 5 and 10 μg/mL. (G) Schematic illustration of SM-CDs surface modifications. (H) FTIR spectra of SM-CDs, CDs-PS, and CDs-PS-Hy. (I) ^1^H-NMR spectra of SM-CDs, CDs-PS, and CDs-PS-Hy. (J) SOD-like activities of CDs-PS and CDs-PS-Hy. (K) FTIR spectra of Re-CDs and Re-CDs-PS-Hy. (L) ^1^H-NMR spectra of Re-CDs and Re-CDs-PS-Hy. (M) SOD-like activities of Re-CDs and Re-CDs-PS-Hy.

The antioxidant enzyme activity of SM-CDs was systematically investigated. First, the total antioxidant capacity of SM-CDs was assessed using ABTS as a chromogenic probe. SM-CDs inhibited the oxidation of ABTS to its blue-green radical cation ABTS•^+^, and the extent of this inhibition was quantified by measuring the absorbance of ABTS•^+^ at 405 nm. The results showed a concentration-dependent increase in the scavenging rate of ABTS•^+^ (Figure 3A). This was visually corroborated by a progressive decrease in its characteristic color. At 100 µg/mL, ABTS•^+^ was nearly eliminated from the reaction system.

Second, the hydroxyl radical (•OH) scavenging capacity of SM-CDs was evaluated. •OH is one of the most reactive and damaging ROS species generated during metabolic processes. The Fenton reaction was used to generate •OH, and TMB served as a colorimetric indicator. SM-CDs suppressed the oxidation of TMB to its oxidized form (oxTMB), which exhibits an absorption peak at 652 nm. The results demonstrated that the scavenging efficiency increased with SM-CDs concentration, reaching approximately 90% at 200 µg/mL (Figure 3B). To further confirm •OH scavenging, DMPO was employed as a spin-trapping agent in combination with electron paramagnetic resonance (EPR) spectroscopy. As shown in Figure 3C, the signal intensity of the DMPO/•OH spin adduct decreased in a dose-dependent manner following the addition of SM-CDs, confirming their •OH scavenging activity.

Third, the SOD-like activity of SM-CDs, which reflects their ability to scavenge •O_2_^-^, was assessed. NBT is reduced by •O_2_^-^ to form blue-purple formazan with an absorbance peak at 560 nm. A concentration-dependent decrease in formazan absorbance was observed upon SM-CDs addition, indicating effective •O_2_^-^ scavenging (Figure 3D). This visual change was also supported by the fading blue color in the reaction solution. At 100 µg/mL, the scavenging efficiency reached 91.97%. The SOD-like enzymatic activity of SM-CDs was further quantified using a commercial WST-1 assay kit and expressed as specific enzyme activity (U/mg). As shown in Figure 3E, the calculated SOD-like activity was 7629.86 U/mg at physiological pH 7.4, which is nearly double that of the natural SOD enzyme 41 and significantly higher than that of *honeysuckle*-derived CDs (HS-CDs), as previously reported 34. To further assess the effect of environmental pH, the SOD-like activity of SM-CDs was also measured under acidic (pH 4.5) and alkaline (pH 8.0) conditions, showing activities of 9056.00 U/mg and 6904.17 U/mg, respectively (Figure S3). These results indicate that SM-CDs maintain strong •O_2_^-^ scavenging capacity across different pH environments relevant to cellular organelles, such as lysosomes and mitochondria. Additionally, EPR spectroscopy using BMPO as a spin-trap for •O_2_^-^ confirmed the strong •O_2_^-^ scavenging performance of SM-CDs. As shown in Figure 3F, the intensity of BMPO/•O_2_^-^ spin adduct signals were markedly reduced upon treatment with 5 or 10 µg/mL SM-CDs. In summary, SM-CDs demonstrate excellent free radical scavenging capabilities, particularly in terms of SOD-like activity. These properties underscore their promising therapeutic potential in ROS-associated pathologies.

Understanding the structure‒activity relationship of nanozymes is of paramount importance for optimizing their catalytic performance. Extensive research has shown that various intrinsic characteristics, such as morphology, size, surface functional groups, and composition, significantly influence nanozyme catalytic activity 42, 43. As previously mentioned, although both SM-CDs and HS-CDs were prepared by the hydrothermal method using traditional Chinese medicine as precursors, the SOD-like activity of SM-CDs was more than twice that of the previously reported HS-CDs 34. While both exhibited uniformly dispersed quasi-spherical nanostructures, their particle size distributions differed significantly. Statistical analysis of TEM images revealed a diameter of 1.83 ± 0.43 nm for SM-CDs, compared to 3.20 ± 0.60 nm for HS-CDs. Notably, the superior catalytic activity of smaller nanoparticles stems from their markedly increased specific surface area, elevated surface atomic ratio, and enhanced surface energy arising from unsaturated bonds, which collectively improve active site accessibility and interfacial reactivity 44. Therefore, the difference in particle size is likely one of the factors contributing to the variation in catalytic activity between these two CDs.

### Roles of surface functional groups in the SOD-like activity of SM-CDs

The surface functional groups of CDs also play an important role in determining their catalytic performance. It has been reported that amino groups on the surface of HS-CDs play a critical role in their SOD-like activity by surface modification and density functional theory (DFT) calculations 34. However, in contrast to HS-CDs, SM-CDs exhibit a lower amino group content (only 4.78% nitrogen as shown in Figure 2I) yet display higher SOD-like activity. This finding prompted us to investigate the role of abundant oxygen-containing functional groups in contributing to the SOD-like activity of SM-CDs. The contributions of carboxyl, carbonyl, and hydroxyl groups were explored through targeted surface modification.

First, 1,3-propanesultone (PS) was used to deactivate the carboxyl and hydroxyl groups. As shown in Figure 3G, PS reacts with surface carboxyl and hydroxyl groups to form esters and ethers, respectively (denoted as CDs-PS), as previously reported 45. Esters can be hydrolyzed under alkaline conditions, while ethers remain stable. Following alkaline hydrolysis in 0.5 M NaOH, the product (CDs-PS-Hy), in which only hydroxyl groups were passivated, was obtained. Fourier-transform infrared (FTIR) spectroscopy revealed dynamic changes during the transition from SM-CDs to CDs-PS and CDs-PS-Hy (Figure 3H). FTIR band assignments and semi-quantitative analysis of functional group changes are shown in Table S1 and Figure S4, respectively. The broad absorption band around 3200 cm^-1^, corresponding to the O-H stretching vibrations of carboxyl and hydroxyl groups, decreased markedly after PS modification and partially recovered after hydrolysis. The 1400 cm^-1^ band, attributed to O-H bending/in-plane vibrations, also exhibited an intensity decrease followed by partial recovery. Meanwhile, the bands at 1200 cm^-1^ (S=O/C-S stretching), 1060 cm^-1^ (S-O stretching), and those at 610 cm^-1^ and 528 cm^-1^ (assigned to -SO_3_^-^), decreased upon hydrolysis of the ester bonds. Additionally, the 1600 cm^-1^ band assigned to C=O stretching vibrations showed a decrease and partial recovery, consistent with the esterification and hydrolysis of carboxyl groups. Complementary to the FTIR results, the ^1^H-nuclear magnetic resonance (^1^H-NMR) spectrum of CDs-PS (Figure 3I) showed new resonances at 2.2 ppm, 3.0 ppm, and 3.5 ppm, corresponding to β-, α-, and γ-hydrogens of -SO_3_^-^, respectively 46. These peaks were attenuated in CDs-PS-Hy, in line with structural changes caused by ester hydrolysis. The SOD-like activities of CDs-PS and CDs-PS-Hy were measured using the WST-1 assay, yielding 426.65 U/mg and 560.87 U/mg, respectively (Figure 3J). Compared to SM-CDs, CDs-PS showed a 94.4% decrease in SOD-like activity, whereas CDs-PS-Hy demonstrated only moderate recovery following carboxyl regeneration. These results show the essential contribution of hydroxyl groups to the SOD-like activity of SM-CDs.

Subsequently, sodium borohydride (NaBH_4_) was used to reduce carbonyl groups to hydroxyl groups on SM-CDs 47, yielding the product denoted as Re-CDs (Figure 3G). Re-CDs were subjected to the same PS passivation and NaOH hydrolysis steps. In this process, the hydroxyl groups were converted to ethers, and the carboxyl groups were esterified and then regenerated, producing Re-CDs-PS-Hy. FTIR analysis confirmed the successful reduction of carbonyl groups to hydroxyl groups in Re-CDs (Figure 3K and Figure S4). The intensities of the 1100 cm^-1^ (C-O stretching vibrations) increased, while the 1600 cm^-1^ band (C=O stretching vibrations) decreased in Re-CDs, confirming carbonyl passivation. Simultaneously, the intensities of the 3200 cm^-1^ (O-H stretching vibrations) and 1400 cm^-1^ (O-H bending/in-plane vibrations) decreased in Re-CDs-PS-Hy, while characteristic -SO_3_^-^ peaks appeared at 1200, 1060, 610, and 528 cm^-1^. The ^1^H NMR spectrum of Re-CDs-PS-Hy also showed new peaks at 2.2 ppm (β-H), 3.0 ppm (α-H), and 3.5 ppm (γ-H), providing direct evidence for -SO_3_⁻ incorporation (Figure 3L). The SOD-like activities of Re-CDs and Re-CDs-PS-Hy were measured as 1062.11 U/mg and 25.30 U/mg, respectively (Figure 3M), corresponding to 86.1% and 99.7% reductions compared to SM-CDs. Despite the conversion of carbonyl groups to hydroxyl groups in Re-CDs, which were previously shown to be critical for SOD-like activity, the pronounced decline in enzyme-like activity suggests that carbonyl groups may play a more dominant role than hydroxyl groups. In contrast, Re-CDs-PS-Hy, which retained only carboxyl groups, exhibited minimal activity. Together with the activity differences observed between CDs-PS and CDs-PS-Hy, these results suggest that carboxyl groups likely play a minor role in the SOD-like activity of SM-CDs. Consistent with previous work, where CDs synthesized from activated carbon exhibited ultrahigh SOD-like activity (1.1 × 10^4^ U/mg), carbonyl groups served as key catalytic sites, whereas hydroxyl groups facilitated reactant binding based on surface modification and DFT calculations 41. Therefore, in alignment with our present findings, carbonyl and hydroxyl functional groups appear to play key roles in the SOD-like activity of SM-CDs.

### Antioxidant and anti-inflammatory effects of SM-CDs in macrophages

The potent ROS scavenging ability of SM-CDs prompted us to investigate their protective effects in myocardial I/R injury. During myocardial I/R injury, excessive ROS activates macrophages and prompts them to transform into the pro-inflammatory M1 phenotype, thereby further amplifying inflammation and oxidative stress. Therefore, we first explored the antioxidant and anti-inflammatory effects of SM-CDs in RAW264.7 cells at the cellular level.

**Figure 4:**
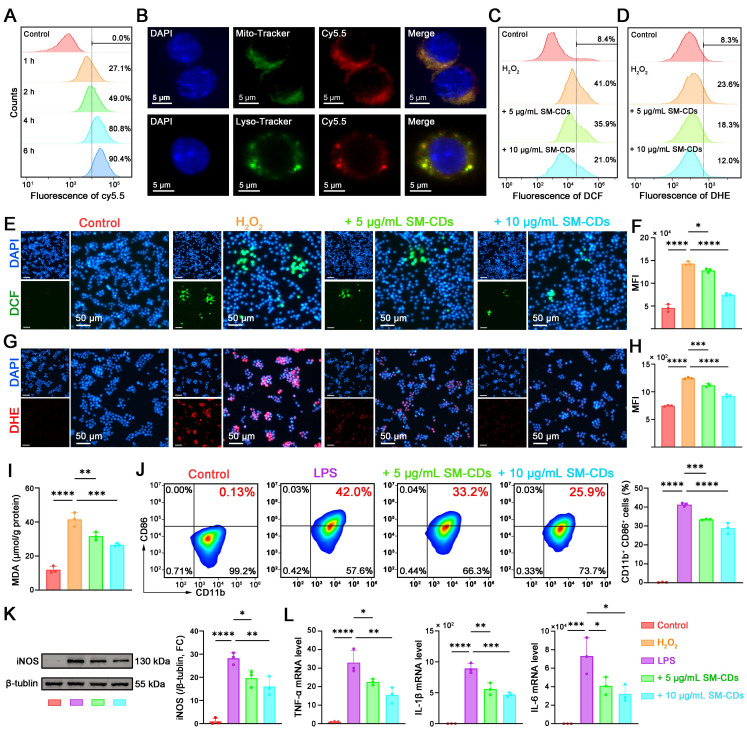
** Antioxidant and anti-inflammatory effects of SM-CDs in macrophages.** (A) Flow cytometry analysis of intracellular uptake of Cy5.5-labeled SM-CDs in RAW264.7 cells. (B) Subcellular localization of SM-CDs in RAW264.7 cells. (C, F) Flow cytometry analysis of intracellular ROS levels. (D, H) Flow cytometry analysis of intracellular •O_2_⁻ levels. (E, G) Representative fluorescent microscopy images of intracellular ROS and •O_2_⁻ in RAW264.7 cells following different treatments. (I) Quantification of cellular MDA levels. (J) Flow cytometry analysis of M1 macrophage polarization (CD11b⁺ CD86⁺) following different treatments. (K) Representative Western blot images and quantification of iNOS protein expression. (L) RT-qPCR analysis of inflammatory cytokines TNF-α, IL-1β, and IL-6. Data are analyzed using One-way ANOVA with Bonferroni post hoc test and represented as mean ± SEM (n=3 for biologically independent samples). *: *P* < 0.05. **: *P* < 0.01. ***: *P* < 0.001. ****: *P* < 0.0001.

The cytotoxicity and biocompatibility of SM-CDs were first assessed in RAW264.7 cells using the CCK-8 assay. The cells were treated with SM-CDs at concentrations ranging from 5 to 100 μg/mL for 24 h. No statistically significant reduction in cell viability was observed at concentrations below 50 μg/mL (Figure S5), indicating that this threshold was safe for subsequent experiments. We next examined the cellular uptake of SM-CDs by RAW264.7 cells. To enable fluorescent detection, SM-CDs were labeled with Sulfo-Cy5.5 amine, which forms covalent bonds with surface carboxyl groups. Flow cytometry revealed a time-dependent increase in uptake by RAW264.7 cells after co-incubation with Cy5.5-labeled SM-CDs, with over 80% of the cells being Cy5.5-positive after 4 h (Figure 4A and Figure S6). To explore subcellular localization, the cells were stained with LysoTracker and MitoTracker following a 4-hour incubation. Fluorescence microscopy showed SM-CDs accumulation in both lysosomes and mitochondria (Figure 4B). Given that mitochondria are the primary sites of ROS production and are highly susceptible to ROS-induced dysfunction, the mitochondrial localization of SM-CDs may contribute to their ability to mitigate oxidative stress at the source.

RAW264.7 cells stimulated with hydrogen peroxide (H_2_O_2_) to induce oxidative stress to assess the antioxidant effect of SM-CDs. CCK-8 assay showed that H_2_O_2_ treatment significantly reduced the number of viable cells, whereas SM-CDs at concentrations of 5-50 μg/mL effectively attenuated this H_2_O_2_-induced cytotoxicity in a dose-dependent manner (Figure S7). Based on these results, 5 and 10 μg/mL SM-CDs were selected for pretreatment in subsequent experiments. Intracellular ROS levels were quantified using 2',7'-dichlorofluorescin diacetate (DCFH-DA), which is hydrolyzed to DCFH within cells and oxidized by ROS into green fluorescent DCF. Fluorescence microscopy revealed strong green fluorescence in H_2_O_2_-treated cells, whereas pre-incubation with SM-CDs significantly reduced fluorescence in a dose-dependent manner (Figure 4E). Quantitative flow cytometry analysis (Figure 4C and Figure 4F) further confirmed that SM-CDs at both tested concentrations significantly suppressed the H_2_O_2_-induced elevation of ROS. To evaluate the ability of SM-CDs to scavenge intracellular •O_2_⁻, we employed dihydroethidium (DHE), a fluorescent probe that reacts with •O_2_⁻ to produce red fluorescence upon binding to nucleic acids. As shown in Figure 4G, pre-incubation with SM-CDs markedly decreased red fluorescence intensity in a concentration-dependent manner. Flow cytometry analysis confirmed this trend (Figure 4D and Figure 4H), demonstrating that both 5 and 10 μg/mL of SM-CDs significantly attenuated H_2_O_2_-induced •O_2_⁻ accumulation. Furthermore, intracellular ROS overproduction triggers lipid peroxidation, leading to elevated MDA levels. As shown in Figure 4I, the MDA content significantly increased in the H_2_O_2_-treated group compared to the control group, whereas SM-CDs treatment substantially reduced this accumulation, which is consistent with its ROS-scavenging function. These findings demonstrate that SM-CDs effectively alleviate oxidative stress in macrophages.

The interplay between oxidative stress and macrophage polarization plays a critical role in the pathological progression of myocardial I/R injury 48. Excess ROS disrupt the balance between pro-inflammatory M1 and anti-inflammatory M2 macrophage phenotypes. Given their potent antioxidant capacity, SM-CDs may help suppress M1 polarization and thereby exert anti-inflammatory effects. To investigate this, lipopolysaccharide (LPS) was used to induce pro-inflammatory polarization. Flow cytometry was conducted using CD11b as a pan-macrophage marker and CD86 as a marker of M1 polarization. LPS stimulation increased the proportion of CD11b⁺ CD86⁺ M1 macrophages to 42%, which was significantly reduced to 33.2% and 25.9% with 5 and 10 μg/mL SM-CDs, respectively (Figure 4J). Western blot analysis further supported this observation, showing that SM-CDs significantly suppressed LPS-induced upregulation of inducible nitric oxide synthase (iNOS), a specific marker of M1 macrophage polarization (Figure 4K, original images in Figure S8). In addition, transcript levels of pro-inflammatory cytokines TNF-α, IL-1β, and IL-6 were markedly increased by LPS, while SM-CDs effectively suppressed their expression at both tested concentrations (Figure 4L). Collectively, these results demonstrate that SM-CDs not only mitigate oxidative stress but also inhibit M1 macrophage polarization, thereby suppressing downstream inflammatory responses.

### Antioxidant and anti-apoptotic effects of SM-CDs in cardiomyocytes

During myocardial I/R injury, excessive ROS not only activate macrophages toward pro-inflammatory M1 polarization, but also directly damage cardiomyocytes, leading to apoptosis. Therefore, we further investigated the antioxidant and anti-apoptotic effects of SM-CDs in cardiomyocytes.

**Figure 5:**
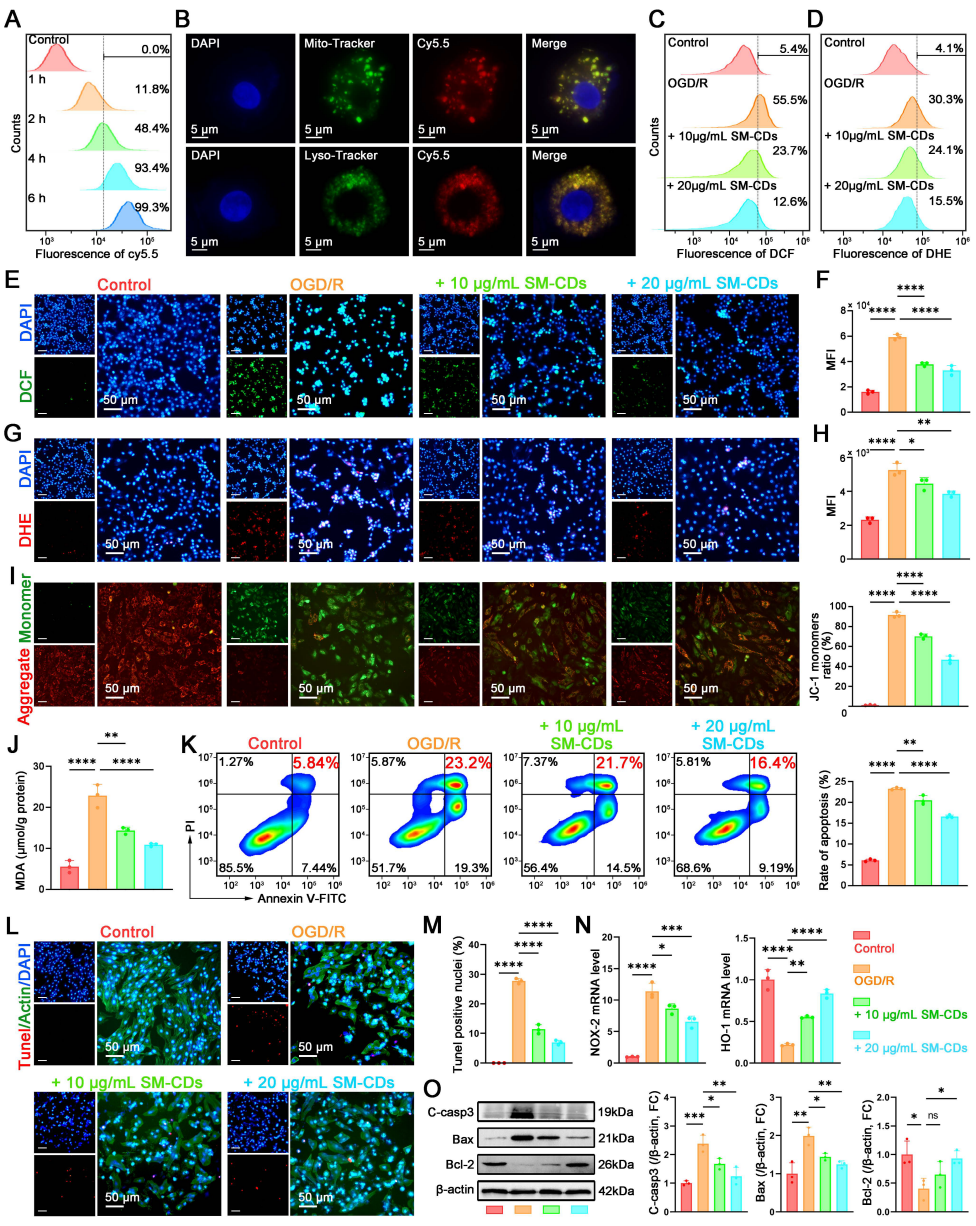
** Antioxidant and anti-apoptotic effects of SM-CDs in cardiomyocytes.** (A) Flow cytometry analysis of Cy5.5-labeled SM-CDs uptake in H9C2 cells. (B) Subcellular localization of SM-CDs in H9C2 cells. (C, F) Flow cytometry analysis of intracellular ROS levels. (D, H) Flow cytometry analysis of intracellular •O_2_⁻ levels. (E, G) Representative fluorescent images of intracellular ROS and •O_2_⁻ scavenging in H9C2 cells following different treatments. (I) Representative JC-1 fluorescent images showing changes in ΔΨm. (J) Quantification of cellular MDA levels. (K) Flow cytometry analysis for the proportion of late apoptotic cells (FITC-Annexin V^+^ PI^+^) following different treatments. (L, M) Representative fluorescent images and quantitative results of cell apoptosis assessed by the TUNEL assay. (N) RT-qPCR analysis of Nox2 (pro-oxidant) and Ho-1 (antioxidant) gene expression in H9C2 cells following different treatments. (O) Representative Western blot images and quantification of cleaved caspase-3, Bax, and Bcl-2. Data are analyzed using One-way ANOVA with Bonferroni post hoc test and represented as mean ± SEM (n=3 for biologically independent samples). *: *P* < 0.05. **: *P* < 0.01. ***: *P* < 0.001. ****: *P* < 0.0001. ns: not significant.

The cytotoxicity and biocompatibility of SM-CDs were also assessed in H9C2 cells to establish a safe concentration range for subsequent experiments. The cells were treated with SM-CDs at concentrations ranging from 5 to 100 μg/mL for 24 h, and viability was evaluated using the CCK-8 assay. The results showed no significant cytotoxicity at concentrations up to 100 μg/mL, confirming excellent biocompatibility of SM-CDs (Figure S9). We then evaluated the cellular uptake of Cy5.5-labeled SM-CDs in H9C2 cells via flow cytometry. Uptake was time-dependent, with over 90% of the cells internalizing SM-CDs after 4 h of incubation (Figure 5A and Figure S10). Fluorescence microscopy following co-staining with Lyso-Tracker and Mito-Tracker confirmed that SM-CDs accumulated not only in lysosomes but also in mitochondria (Figure 5B), similar to findings in RAW macrophages. This mitochondrial localization enhances the ROS-scavenging potential of SM-CDs, mitigating oxidative damage at their primary source. To mimic myocardial I/R injury *in vitro*, we subjected H9C2 cells to a process of oxygen-glucose deprivation/reperfusion (OGD/R). The cells underwent 6 h of OGD followed by reoxygenation in the presence of SM-CDs (5-100 μg/mL). After a 12-hour recovery, CCK-8 assays revealed that OGD/R significantly reduced the number of viable cells, while SM-CDs (10-100 μg/mL) dose-dependently attenuated this damage (Figure S11). Based on both efficacy and safety, concentrations of 10 and 20 μg/mL were selected for subsequent experiments. Fluorescence microscopy and flow cytometry analyses demonstrated that OGD/R significantly increased the intracellular ROS (DCFH-DA probe) and •O_2_⁻ (DHE probe) levels in H9C2 cells. SM-CDs significantly reduced both markers in a dose-dependent manner (Figure 5C-H). Furthermore, lipid peroxidation, assessed via MDA levels, was markedly elevated after OGD/R but significantly reduced by SM-CDs treatment (Figure 5J), underscoring the ROS-scavenging ability of SM-CDs in cardiomyocytes.

Given that oxidative stress-induced ROS is a key trigger of apoptosis via mitochondrial dysfunction and DNA damage, we next explored the anti-apoptotic effects of SM-CDs. Mitochondrial membrane potential (ΔΨm) depolarization, a hallmark of early apoptosis, was first assessed using the JC-1 fluorescent probe. JC-1 forms red fluorescent J-aggregates in polarized mitochondria (high ΔΨm) but shifts to green monomers upon depolarization (low ΔΨm), with the green/red fluorescence ratio serving as a quantitative indicator of apoptosis initiation. As demonstrated in Figure 5I, SM-CDs effectively attenuated the OGD/R-induced ΔΨm collapse. Moreover, SM-CDs partially restored the ATP production levels reduced by OGD/R treatment (Figure S12), collectively indicating the stabilization of mitochondrial function. Then, apoptotic cell populations were analyzed by flow cytometry using FITC-Annexin V to label early apoptotic cells and propidium iodide (PI) to identify late apoptotic or necrotic cells. The second quadrant (FITC-Annexin V-positive/PI-positive) represents late apoptotic cells, while the fourth quadrant (FITC-Annexin V-positive/PI-negative) corresponds to early apoptotic populations. The results demonstrate that SM-CDs significantly attenuated OGD/R-induced apoptosis, evidenced by the reduced proportions of both early and late apoptotic cells (Figure 5K). Besides, TUNEL assay was employed to further evaluate the anti-apoptotic effects. Under fluorescence microscopy, apoptotic cells exhibited distinct red fluorescence, confirming DNA fragmentation characteristic of programmed cell death. Consistent with the above results, OGD/R treatment significantly exacerbated apoptosis, whereas SM-CDs markedly attenuated this process by reducing the proportion of TUNEL-positive cells (Figure 5L-M). Additionally, we quantified oxidative stress-related genes. SM-CDs downregulated the pro-oxidant gene Nox2 while upregulating the antioxidant gene Ho-1 (Figure 5N). Western blot analysis further showed that OGD/R markedly increased pro-apoptotic proteins (cleaved caspase-3, Bax) and decreased the anti-apoptotic protein Bcl-2. SM-CDs reversed these trends, promoting cell survival (Figure 5O, original images in Figure S13). Collectively, these findings demonstrate that SM-CDs confer cardioprotective effects through dual mechanisms: potent antioxidant activity via ROS scavenging and anti-apoptotic efficacy by preserving mitochondrial integrity and modulating key apoptotic regulators.

### *In vivo* therapeutic effects of SM-CDs on myocardial I/R injury

**Figure 6:**
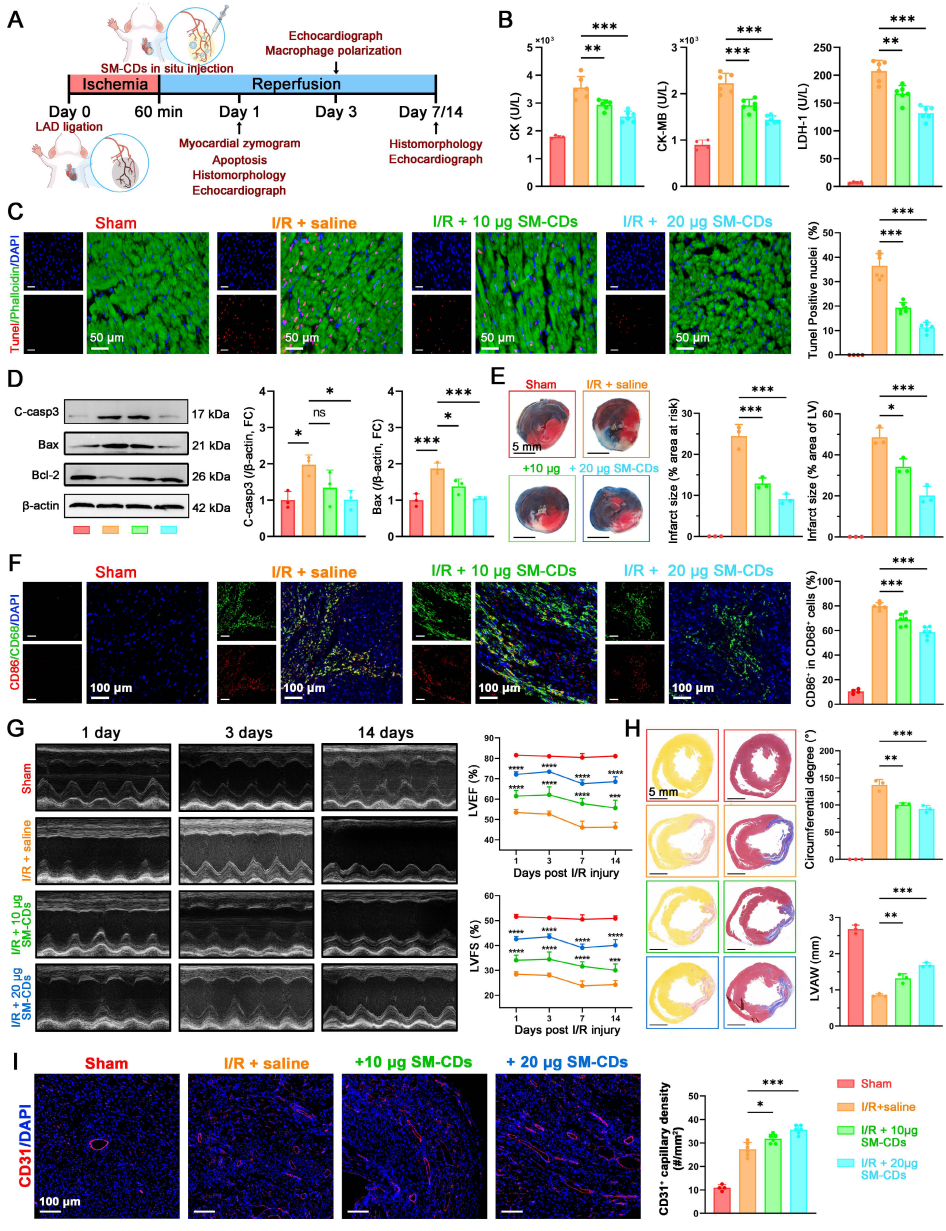
***In vivo* therapeutic effects of SM-CDs in a rat model of myocardial I/R injury.** (A) Schematic illustration of the animal treatment protocol. (B) Serum levels of cardiac enzymes CK, CK-MB, and LDH-1. (C) Representative TUNEL staining images and quantification of apoptotic cardiomyocytes. (D) Representative Western blot images and quantification of cleaved caspase-3 and Bax (Bcl-2 results shown in Figure S16). (E) Representative TTC-Evans blue dual stained heart slices and quantification of infarct size. (F) Representative fluorescent images and quantification analysis of M1 macrophage polarization (CD68^+^ CD86^+^) in ischemic region. (G) Representative echocardiographic images and quantification analysis for LVEF and LVFS on days 1, 3, 7 and 14 post-reperfusion (statistical comparisons were made between the I/R+Saline and I/R+SM-CDs group). (H) Representative picrosirius red and Masson's trichrome stained heart slices and quantification of myocardial fibrosis on day 14 post-reperfusion. (I) Representative fluorescent images and quantification of CD31^+^ capillary in the ischemic region on day 14 post-reperfusion. Data are analyzed using One-way ANOVA with Bonferroni post hoc test and represented as mean ± SEM. In b-c, f, and i, n = 4 biologically independent animals for Sham groups and 6 for I/R groups. In d-e and h, n = 3 biologically independent animals. In g, n = 6 for biologically independent animals. *: *P* < 0.05. **: *P* < 0.01. ***: *P* < 0.001. ****: *P* < 0.0001. ns: not significant.

Given the inspiring antioxidant, anti-inflammatory, and anti-apoptotic effects of SM-CDs observed *in vitro*, we further investigated the therapeutic potential in a rat model of myocardial I/R injury. The model was established by ligation of the left anterior descending (LAD) coronary artery for 60 min, followed by reperfusion. Representative electrocardiograms confirming myocardial ischemia during ligation are shown in Figure S14. SM-CDs were locally administered into the ischemic anterior wall of the left ventricle immediately upon reperfusion. The therapeutic effects were comprehensively assessed across multiple indices at predefined time points, as described in the experimental workflow (Figure 6A).

At 24 h post-reperfusion, the serum levels of cardiac injury biomarkers including creatine kinase (CK), CK-MB, and lactate dehydrogenase-1 (LDH-1) were significantly elevated in the I/R group. Notably, SM-CDs treatment markedly reduced the levels of all three enzymes, indicating substantial mitigation of myocardial injury (Figure 6B). To evaluate mitochondrial function *in vivo*, the ΔΨm and ATP production levels in cardiac tissue were examined. As shown in Figure S15, I/R injury resulted in a marked loss of ΔΨm and ATP levels, whereas SM-CDs treatment effectively restored ΔΨm and ATP generation, suggesting improved mitochondrial function and energy metabolism. Apoptosis within myocardial tissues was assessed via TUNEL staining and fluorescence microscopy, revealing a significant increase in TUNEL-positive cardiomyocytes in the I/R group relative to the sham group. In contrast, SM-CDs treatment significantly suppressed apoptosis, with higher SM-CDs doses yielding more pronounced anti-apoptotic effects (Figure 6C). These findings were further supported by Western blot analysis, which revealed that SM-CDs downregulated pro-apoptotic proteins cleaved caspase-3 and Bax, while upregulating the anti-apoptotic protein Bcl-2 (Figure 6D and Figure S16, original images in Figure S17). Given the clinical relevance of infarct size as a prognostic indicator in myocardial I/R injury 49, we evaluated infarct size using 2,3,5-triphenyltetrazolium chloride (TTC)-Evans blue dual staining. Non-ischemic areas were stained blue by Evans blue, ischemic but non-infarct regions were stained red by TTC, and infarct areas remained unstained (white). The infarct size ratio, calculated as the percentage of infarct area relative to the area at risk (infarct + ischemic regions) and also relative to the total left ventricular area, served as a key metric of I/R injury severity. The results demonstrated that SM-CDs treatment significantly reduced infarct size across both metrics, indicating its efficacy in limiting myocardial damage (Figure 6E). Since myocardial inflammation typically peaks around 72 h post-I/R, we evaluated macrophage polarization at this time point by immunostaining for CD68 (pan-macrophage marker) and CD86 (M1 macrophage marker). SM-CDs treatment notably reduced the percentage of CD86^+^ macrophages, suggesting that SM-CDs suppress pro-inflammatory M1 polarization and thereby alleviate inflammatory responses (Figure 6F).

To assess cardiac functional recovery, echocardiography was performed on days 1, 3, 7 and 14 following reperfusion. At all-time points, SM-CDs-treated rats presented significantly improved cardiac performance (Figure 6G and Figure S18). This was evidenced by higher LVEF and LVFS values compared to untreated I/R controls, confirming the sustained cardioprotective benefits of SM-CDs. During cardiac repair following myocardial I/R injury, the formation of neovascularized granulation tissue and fibrotic remodeling of necrotic myocardium constitute critical determinants of cardiac functional recovery 50. Suppression of excessive inflammatory responses facilitates vascular neovascularization and promotes granulation tissue formation. While moderate fibrosis is essential for maintaining cardiac structural integrity, excessive fibrotic deposition leads to myocardial stiffness and functional impairment. Picrosirius red and Masson's trichrome staining were performed to evaluate myocardial fibrosis, while CD31 immunofluorescence staining were employed to access capillary density. SM-CDs reduced fibrotic region, as reflected by a decreased circumferential degree of fibrosis, and partially preserved anterior wall thickness (Figure 6H). Additionally, SM-CDs treatment significantly increased the CD31^+^ capillary density at both 7 and 14 days after I/R injury (Figure 6I and Figure S19), indicating improved neovascularization. These results suggest that SM-CDs promote vascular remodeling and myocardial repair through multifaceted mechanisms, including attenuation of oxidative stress, inflammation, and apoptosis. Improved microvascular perfusion likely facilitates nutrient and oxygen delivery to the ischemic myocardium, thereby limiting necrosis and reducing pathological fibroblast activation. This process ultimately prevents excessive collagen accumulation and preserves myocardial elasticity.

In summary, the *in vivo* studies clearly demonstrate that SM-CDs confer significant cardioprotection against myocardial I/R injury by modulating cell death, inflammation, oxidative stress, and tissue remodeling. These findings highlight the therapeutic potential of SM-CDs as a multifunctional nanotherapeutic agent for myocardial I/R injury.

Intramyocardial injection was adopted in this study to ensure accurate local delivery and consistent dosing in the rat I/R model. However, apart from applications such as cardiac transplantation, this approach has limited clinical applicability. For translational purposes, systemic administration routes such as intravenous injection or intracoronary infusion via catheterization may be more practical, as these are compatible with current interventional cardiology procedures. Moreover, active cardiac targeting designs, including surface modification with polypeptides (e.g., cardiac targeting peptides 51, atrial natriuretic peptide 52) or biomolecules (e.g., mannan 20, tannic acid 53), could enhance the selective uptake of SM-CDs by ischemic myocardium and minimize off-target distribution. Under these circumstances, comprehensive pharmacokinetic and biodistribution studies will be further conducted to evaluate the *in vivo* circulation time, clearance pathways, and cardiac accumulation of SM-CDs. These improvements will help bridge the gap between experimental findings and potential clinical translation.

### Transcriptomic profiling reveals the therapeutic mechanisms of SM-CDs in myocardial I/R injury

**Figure 7:**
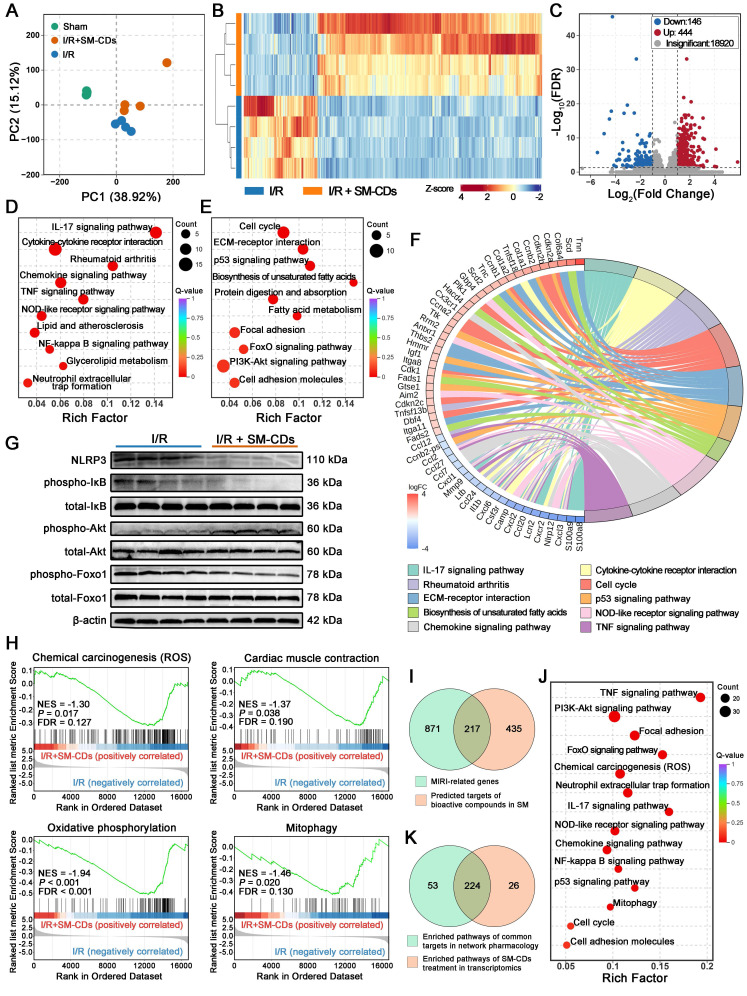
** Transcriptomic analysis revealing the therapeutic mechanisms of SM-CDs in myocardial I/R injury.** (A) PCA score plot of gene expression profiles in the sham, I/R, and I/R+SM-CDs groups. (B) Heatmap showing distinct gene expression patterns between I/R and I/R+SM-CDs groups. (C) Volcano plot of DEGs between I/R and I/R+SM-CDs groups (criteria: |fold change| > 2 and FDR < 0.05). (D) KEGG pathway enrichment analysis of down-regulated DEGs in the I/R+SM-CDs group compared to the I/R group. (E) KEGG pathway enrichment analysis of up-regulated DEGs in the I/R+SM-CDs group compared to the I/R group. (F) Chord diagram showing top 10 enriched KEGG pathways and their corresponding associated genes. (G) Representative Western blot images of NLRP3, p-IκB, total-IκB, p-Akt, total Akt, p-Foxo1, and total Foxo1. (H) GSEA for gene sets in chemical carcinogenesis (ROS), cardiac muscle contraction, oxidative phosphorylation, and mitophagy in the I/R+SM-CDs group compared to the I/R group. (I) Venn diagram illustrating the overlap between *Salvia miltiorrhiza* targets identified by network pharmacology and myocardial I/R injury-related genes. (J) KEGG pathway enrichment analysis of the overlapping genes between *Salvia miltiorrhiza* targets and I/R-related genes. (K) Venn diagram illustrating the overlap between KEGG pathways enriched from SM-CDs-induced DEGs and those enriched from network pharmacology-screened overlapping genes.

To further elucidate the cardioprotective mechanisms of SM-CDs *in vivo*, transcriptomic profiling was performed on myocardial tissues from the ischemic zone collected 24 h post-reperfusion. Principal component analysis (PCA) revealed distinct transcriptional signatures among the sham group, I/R injury group, and SM-CDs-treated I/R group (Figure 7A). A hierarchical clustering heatmap further demonstrated that SM-CDs administration substantially altered gene expression patterns in the ischemic myocardium of I/R rats (Figure 7B). Based on the thresholds of |fold change| > 2 and false discovery rate (FDR) < 0.05, a total of 590 differentially expressed genes (DEGs) were identified. Volcano plot analysis showed 444 upregulated and 146 downregulated DEGs in the SM-CDs-treated group compared to the untreated I/R group (Figure 7C). Kyoto Encyclopedia of Genes and Genomes (KEGG) pathway enrichment analysis was performed to investigate the biological implications of these DEGs. The downregulated DEGs were predominantly associated with inflammatory signaling pathways, including the IL-7, chemokine, TNF, NOD-like receptor, and NF-κB pathways (Figure 7D). In contrast, the upregulated DEGs were significantly enriched in pathways related to cell cycle regulation (e.g., cell cycle, p53, and PI3K-Akt signaling), energy metabolism (e.g., FoxO signaling and biosynthesis of unsaturated fatty acids), and tissue repair (e.g., ECM-receptor interaction, focal adhesion, and cell adhesion molecules) (Figure 7E). A KEGG chord diagram summarizing the top 10 enriched pathways and their corresponding DEGs is presented in Figure 7F. The expression of pro-inflammatory mediators, such as S100A8/A9 and multiple chemokines from the CC and CXC subfamilies, was significantly downregulated in the SM-CDs group. In contrast, genes involved in cell cycle progression, including cyclin A (CCNA), cyclin B (CCNB), and cyclin-dependent kinase inhibitors (CDKN), were markedly upregulated. Gene Ontology (GO) enrichment analysis supported these findings, with downregulated DEGs enriched in inflammatory response terms and upregulated DEGs associated with processes related to cell proliferation and regeneration (Figure S20-S21).

Integrating these transcriptomic results, we suppose that SM-CDs may exert cardioprotective effects by mitigating oxidative stress, suppressing inflammatory signaling cascades, and promoting metabolic adaptation and tissue repair. These therapeutic effects are likely mediated through coordinated regulation of four key signaling pathways: the NF-κB signaling pathway, the NOD-like receptor (NLR) signaling pathway, the PI3K-Akt signaling pathway, and the FoxO signaling pathway.

The NF-κB signaling pathway is a transcription factor-mediated cascade extensively involved in key physiological processes, including inflammation, immune regulation, and apoptosis. During myocardial I/R injury, excessive production of ROS and pro-inflammatory cytokines activate the IκB kinase (IKK) complex, leading to phosphorylation and subsequent degradation of IκB proteins 54. This degradation releases NF-κB dimers, which translocate into the nucleus and initiate the transcription of pro-inflammatory genes, thereby amplifying inflammatory responses and promoting apoptosis. In parallel, damage-associated molecular patterns (DAMPs) generated during I/R injury such as ROS, ATP depletion, and organelle stress trigger the NLR signaling pathway, mainly through NLRP3 inflammasome assembly 55. This activation promotes the secretion of pro-inflammatory cytokines, notably IL-1β and IL-18, which further exacerbate cardiomyocyte injury and apoptotic cell death. Importantly, the NF-κB and NLR signaling pathways exhibit synergistic crosstalk: NF-κB activation enhances NLRP3 expression, while inflammasome-mediated cytokine release further stimulates NF-κB activity 56. This bidirectional interaction forms a feedforward loop that intensifies inflammation and aggravates myocardial damage. By scavenging ROS and attenuating inflammatory stimuli, SM-CDs may disrupt this pathological loop and suppress the overactivation of both NF-κB and NLR signaling pathways. To confirm this hypothesis, Western blot analysis was performed to assess the levels of total IκB, phosphorylated IκB (p-IκB), and NLRP3 (Figure 7G and Figure S22, original images in Figure S23). The observed decrease in the p-IκB/total-IκB ratio, along with reduced NLRP3 expression, indicated that SM-CDs effectively downregulate NF-κB activation and NLRP3 inflammasome signaling. The results suggest that SM-CDs mitigate myocardial I/R injury by concurrently inhibiting the NF-κB and NLR signaling pathways, thereby suppressing inflammatory cascades and reducing cardiomyocyte apoptosis.

The PI3K-Akt signaling pathway is a critical and multifaceted transduction cascade involved in cellular survival, proliferation, metabolism, and apoptosis. Upon activation by extracellular signals, phosphoinositide 3-kinase (PI3K) catalyzes the formation of phosphatidylinositol (3,4,5)-trisphosphate (PIP3), which recruits Akt to the membrane and facilitates its activation via phosphorylation. Activated Akt (p-Akt) subsequently regulates a wide array of downstream effectors to modulate diverse cellular functions. In the context of myocardial I/R injury, extensive evidence supports the cardioprotective role of PI3K-Akt signaling by attenuating inflammation, suppressing oxidative stress, preventing apoptosis, and promoting cell survival 57. Under pathological conditions, ROS-induced oxidative damage can impair PI3K-Akt signaling. However, ROS scavenging may relieve this suppression while preserving the oxidative inactivation of phosphatase and tensin homolog (PTEN), a negative regulator that antagonizes PI3K-Akt activity by dephosphorylating PIP3. This dual mechanism promotes PIP3 accumulation and enhances Akt activation. Western blot analysis of the p-Akt/total Akt ratio, a key indicator of PI3K-Akt pathway activity, showed a significant increase in SM-CDs-treated groups compared to the untreated I/R group (Figure 7G and Figure S22), indicating that SM-CDs enhance PI3K-Akt signaling. These results suggest that the SM-CDs also exert cardioprotective effects against myocardial I/R injury by enhancing PI3K-Akt signaling activity.

The FoxO signaling pathway, which centers on FoxO family transcription factors, plays essential roles in regulating apoptosis, oxidative stress responses, and metabolic homeostasis 58. Previous studies have shown that FoxO activation supports cardiomyocyte survival during myocardial I/R injury 59. The transcriptional activity of FoxOs is primarily governed by their phosphorylation status: unphosphorylated FoxOs translocate to the nucleus and activate target gene expression, while phosphorylation leads to nuclear exclusion, subsequent ubiquitin-mediated degradation, and loss of transcriptional activity. Western blot analysis demonstrated a significant decrease in the phosphorylated Foxo1 (p-Foxo1)/total Foxo1 ratio in SM-CDs-treated groups relative to the I/R group (Figure 7G and Figure S22), indicating enhanced activation of the FoxO signaling pathway. This suggests that SM-CDs may exert cardioprotective effects by promoting FoxO-mediated antioxidant responses, apoptosis inhibition, and metabolic adaptation. Notably, although classical PI3K-Akt signaling is known to inactivate FoxOs via phosphorylation, our data revealed a paradoxical reduction in Foxo1 phosphorylation despite Akt activation. This apparent contradiction may be explained by SM-CDs' capacity to relieve intracellular oxidative stress or to engage alternative FoxO-activating mechanisms that override Akt-mediated phosphorylation-dependent suppression.

Besides, gene set enrichment analysis (GSEA) was employed to access expression changes in predefined gene sets. In addition to the previously described signaling pathways, SM-CDs treatment led to significant suppression of four key pathways in SM-CDs-treated groups compared to the I/R injury group: chemical carcinogenesis (ROS), cardiac muscle contraction, oxidative phosphorylation, and mitophagy (Figure 7H). These findings suggest that SM-CDs may also exert cardioprotective effects through (1) scavenging excessive ROS, thereby attenuating the ROS-mediated activation of downstream inflammatory and apoptotic signaling pathways; (2) suppressing dysregulated myocardial contraction and hypercontractile stress under I/R conditions, thus alleviating secondary injury caused by calcium overload and metabolic dysfunction; (3) reducing hyperactive oxidative phosphorylation during I/R to minimize additional ROS generation; and (4) inhibiting excessive mitophagy to preserve mitochondrial integrity and homeostasis. In conclusion, these results indicate that SM-CDs not only directly eliminate ROS but also regulate signaling pathways involved in energy metabolism, mitochondrial function, and autophagy, thereby providing comprehensive cardioprotective against myocardial I/R injury.

### Network pharmacology analysis links *Salvia miltiorrhiza*'s bioactivity to the therapeutic effects of SM-CDs

SM-CDs inherently possess ROS-scavenging ability due to their structural properties, thereby mediating downstream anti-inflammatory and anti-apoptotic effects. However, transcriptomic profiling, pathway enrichment, and GSEA results suggest that the cardioprotective effects of SM-CDs against myocardial I/R injury involve additional mechanisms beyond redox regulation. We hypothesize that the use of *Salvia miltiorrhiza* as a carbon precursor may endow SM-CDs with specific surface chemical moieties derived from the herb, potentially retaining its inherent pharmacological properties. Numerous studies have reported the cardioprotective effects of *Salvia miltiorrhiza* and its major bioactive components (e.g., tanshinones and salvianolic acids) in myocardial I/R injury 35, particularly through modulation of the NF-κB, NLR, PI3K-Akt, and FoxO signaling pathways mentioned above 60-63. This suggests that SM-CDs may integrate both the ROS-scavenging functionality of carbon nanomaterials and the multi-target regulatory activity of the herbal precursor.

Although direct experimental confirmation remains challenging, we performed a preliminary network pharmacology analysis to compare the signaling pathways affected by SM-CDs with the predicted molecular mechanisms of *Salvia miltiorrhiza* in the context of myocardial I/R injury (Figure 7I-K). A total of 652 target genes associated with *Salvia miltiorrhiza* bioactive components were identified from the TCMSP, SwissADME, and SwissTargetPrediction databases. Simultaneously, 1,088 myocardial I/R-related genes were collected from GeneCards, OMIM, and DisGeNET databases. Intersection analysis revealed 217 overlapping genes between the herb targets and I/R-associated genes. Subsequent KEGG enrichment analysis of these 217 targets yielded 277 signaling pathways. Notably, among the 250 pathways enriched in our SM-CDs transcriptomic data, 224 (89.6%) overlapped with the 277 pathways predicted for *Salvia miltiorrhiza*, demonstrating a remarkable mechanistic concordance. This high degree of overlap supports the hypothesis that SM-CDs retain substantial pharmacological features from their *Salvia miltiorrhiza* precursor, contributing to their multifaceted cardioprotective actions in myocardial I/R injury.

### *In vivo* biosafety assessment of SM-CDs

**Figure 8:**
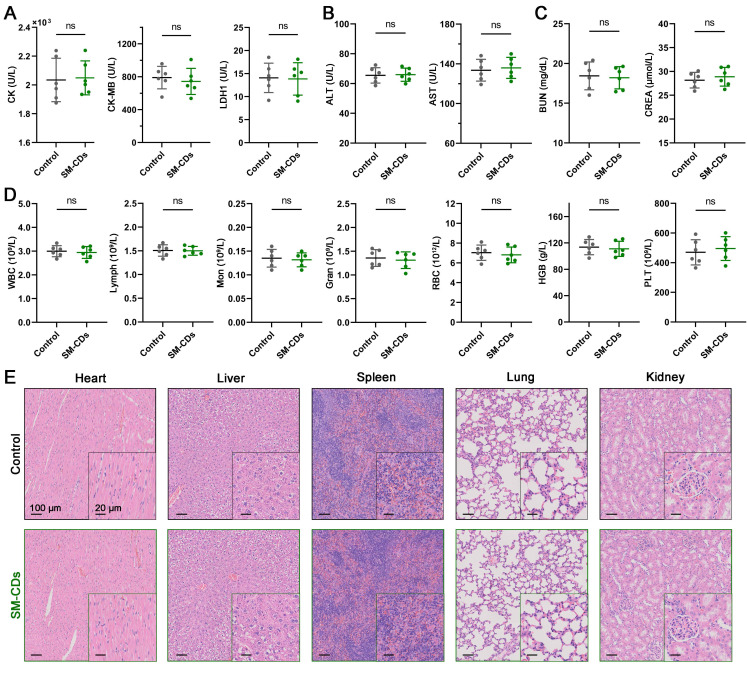
***In vivo* biosafety assessment of SM-CDs.** (A) Serum levels of cardiac enzymes CK, CK-MB, and LDH-1 on day 1 after SM-CDs administration. (B) Serum levels of liver function indices ALT and AST on day 14 after SM-CDs administration. (C) Serum levels of renal function indices BUN and CREA on day 14 after SM-CDs administration. (D) Complete blood count results on day 14 after SM-CDs administration. Data are analyzed using two-tailed unpaired Student *t*-test and represented as mean ± SEM (n=6 for biologically independent animals). ns: not significant. (E) Representative H&E-stained images of major organs collected on day 14 after SM-CDs administration.

Biosafety is a critical prerequisite for the clinical translation of carbon dot nanozymes like SM-CDs 64, 65. Although prior *in vitro* studies confirmed their biocompatibility, we further evaluated their *in vivo* biosafety using the same intramyocardial injection protocol employed in the myocardial I/R injury model. Specifically, a bolus of 10 μL SM-CDs (4 mg/mL, twice the high therapeutic dose) was directly administered into the left ventricular anterior wall. Over a 14-day observation period, SM-CDs-treated rats exhibited normal weight gain, comparable to that of the controls (Figure S24). Serum biochemical analysis on days 1, 7, and 14 post-injection revealed no significant differences in cardiac injury markers (CK, CK-MB, LDH-1), liver function indices (ALT, AST), or renal function indices (BUN, CREA) between the SM-CDs-treated and control groups, with all values remaining within physiological standards (Figure 8A-D and Figure S25). Moreover, HE-stained tissue sections of major organs collected on day 7 and 14 post-injection revealed no signs of inflammation, necrosis, fibrosis, or hemorrhage (Figure 8E and Figure S25). Collectively, these results demonstrate that SM-CDs exhibit excellent *in vivo* biosafety and systemic biocompatibility at the tested therapeutic dose, supporting their potential for further translational development.

## Conclusion

In this study, we developed a green and facile hydrothermal method to synthesize carbon dot nanozymes (SM-CDs) using *Salvia miltiorrhiza* as a natural carbon precursor. The resulting SM-CDs featured ultrasmall size and abundant surface oxygen-containing groups, exhibiting potent antioxidant enzymatic activity—particularly an exceptionally high SOD-like activity nearly double that of natural SOD. Surface modification studies suggested that carbonyl and hydroxyl groups play crucial roles in this catalytic performance. *In vitro*, SM-CDs efficiently scavenged intracellular ROS, inhibited pro-inflammatory M1 macrophage polarization, and reduced cardiomyocyte apoptosis. *In vivo*, intramyocardial administration of SM-CDs in a rat myocardial I/R model significantly attenuated inflammation, apoptosis, and infarct size, while promoting cardiac repair by reducing fibrosis, enhancing neovascularization, and improving cardiac function. Transcriptomic analysis revealed that SM-CDs confer cardioprotection through multiple pathways, including oxidative stress mitigation, inflammatory suppression, and tissue remodeling, potentially mediated via the inhibition of NF-κB and NLR signaling and the activation of PI3K-Akt and FoxO pathways. Network pharmacology further suggested that SM-CDs may retain bioactive characteristics derived from *Salvia miltiorrhiza*. Comprehensive biosafety evaluations confirmed excellent biocompatibility with no systemic toxicity. Together, these findings highlight the promise of SM-CDs as a novel, biocompatible, and multifunctional nanozyme for the treatment of myocardial I/R injury and potentially other oxidative stress-related disorders. Future studies will focus on improving the cardiac targeting capability of SM-CDs and further elucidating their long-term therapeutic efficacy and pharmacokinetics.

## Methods

### Synthesis of SM-CDs

SM-CDs were synthesized via a hydrothermal method using *Salvia miltiorrhiza* as the carbon precursor. Briefly, 2.5 g of pulverized *Salvia miltiorrhiza* roots were homogenized in 50 mL of ultrapure water under stirring. The resulting suspension was transferred into a Teflon-lined stainless-steel autoclave and heated at 240 °C for 12 h. After natural cooling, the solution was centrifuged and filtered through a 0.22 μm membrane. The filtrate was dialyzed (1 kDa MWCO) against ultrapure water for 72 h with water changes every 12 h. The final SM-CDs product was lyophilized and stored for further use.

### Characterization of SM-CDs

The morphology of SM-CDs was characterized by high-resolution HR-TEM and AFM. UV-vis absorption and fluorescence excitation/emission spectra were recorded using a spectrophotometer and a fluorescence spectrometer, respectively. The elemental composition and chemical structures were analyzed using XPS. Surface functional groups were further identified by FTIR spectroscopy and ^1^H-NMR spectroscopy. Instrumental parameters and characterization methods are detailed in the Supplementary Information.

### Assessment of antioxidant enzymatic activity

The antioxidant capacity of SM-CDs was evaluated by assessing their scavenging activities against ABTS•⁺, •OH, and •O_2_^-^. The ABTS•⁺ scavenging was quantified at 405 nm using a commercial assay kit. The •OH scavenging activity was measured via a TMB-based colorimetric assay and confirmed by EPR spectroscopy using DMPO as a spin-trapping agent. The •O_2_^-^ scavenging capability was assessed by NBT reduction, a WST-1-based SOD assay kit, and EPR with BMPO as a spin-trapping probe. Detailed protocols and reagent information are described in the Supplementary Information.

### Surface modification of SM-CDs

To investigate the contribution of surface functional groups to the antioxidant activity of SM-CDs, a series of chemical modifications were performed targeting carboxyl, carbonyl, and hydroxyl groups. Carboxyl and hydroxyl groups were modified using (PS to form ester and ether linkages (CDs-PS), followed by alkaline hydrolysis to regenerate carboxyl groups and selectively block hydroxyl groups (CDs-PS-Hy). Carbonyl groups were reduced to hydroxyl groups using NaBH_4_ to generate Re-CDs, which were further processed to Re-CDs-PS-Hy via the same PS modification route. The structural changes were characterized by FTIR and ^1^H-NMR spectroscopy. Their antioxidant capacity was further quantified by measuring SOD-like activity using the WST-1-based SOD assay kit. Detailed synthetic protocols are provided in the Supplementary Information.

### *In vitro* cellular experiments

The mouse macrophage cell line RAW264.7 (RRID: CVCL_0493) and rat cardiomyocyte cell line H9C2 (RRID: CVCL_0286) were purchased from the China Center for Type Culture Collection (CCTCC, Wuhan, China). Both cell lines were authenticated by the supplier and tested negative for mycoplasma contamination prior to experimentation. Cell lines were cultured in high-glucose DMEM supplemented with 10% fetal bovine serum and 1% penicillin-streptomycin in a humidified incubator at 37 °C with 5% CO_2_. To assess cytotoxicity and biocompatibility, both cell lines were exposed to varying concentrations of SM-CDs for 24 h, and cell viability was determined using a commercial CCK-8 assay kit according to the manufacturer's protocol. For cellular uptake studies, SM-CDs were labeled with Cy5.5 and tracked via flow cytometry and confocal microscopy. In oxidative stress experiments, macrophages were stimulated with H_2_O_2_ to induce intracellular ROS production, and cardiomyocytes were subjected to oxygen-glucose deprivation for 4 h followed by 12 h reoxygenation to simulate myocardial I/R injury in vitro. ROS and •O_2_^-^ levels were assessed using DCFH-DA and DHE fluorescent probes, respectively. Flow cytometry and fluorescence microscopy were used for quantification and visualization. For anti-inflammatory evaluation in RAW264.7 cells, LPS was used to induce M1 macrophage polarization. The proportion of M1 phenotype (CD11b⁺ CD86⁺) was quantified by flow cytometry, and the expression of M1 marker iNOS was detected by Western blot. mRNA levels of pro-inflammatory cytokines (TNF-α, IL-1β, IL-6) were determined via RT-qPCR. In H9C2 cardiomyocytes, apoptosis was evaluated after OGD/R and SM-CDs treatment. ΔΨm was measured using JC-1 staining, and the ATP production level was also determined to evaluate mitochondrial function. Late apoptotic cells were identified by Annexin V-FITC/PI dual staining followed by flow cytometry. DNA fragmentation was assessed by TUNEL assay under fluorescence microscopy. Detailed protocols and reagent information are provided in the Supplementary Information.

### Rat model of myocardial I/R injury

All experimental procedures involving animals were conducted in accordance with the ethical guidelines approved by the Animal Ethics Committee of Xi'an Jiaotong University and complied with the protocols of the Institutional Animal Care and Use Committee (IACUC) of the university (No: XJTUAE2024-785). Male Sprague-Dawley rats (230-270 g) were anesthetized and subjected to LAD coronary artery ligation for 60 min, followed by reperfusion. SM-CDs (2 mg/mL, 5 μL or 10 μL) were intramyocardially injected into the ischemic border zone at the onset of reperfusion. Blood and tissue samples were collected at designated time points. Serum levels of CK, CK-MB, and LDH-1 were measured to assess cardiac injury. The ΔΨm and ATP production levels in cardiac tissue were examined to evaluate mitochondrial function *in vivo*. For *in vivo* anti-apoptotic assessment, TUNEL staining was performed to detect apoptotic cardiomyocytes, and Western blotting was conducted to quantify the expression of apoptosis-related proteins (cleaved caspase-3, Bax, and Bcl-2). Myocardial infarct size was evaluated by TTC and Evans blue dual staining. Echocardiography was performed at 1, 3, and 7 days post-reperfusion to assess cardiac function. Myocardial fibrosis and angiogenesis were assessed by Masson's trichrome staining and CD31 immunohistochemistry, respectively. Detailed surgical procedures, echocardiographic settings, and staining protocols are provided in the Supplementary Information.

### Transcriptomic and pathway analysis

Ischemic myocardial tissues were harvested at 24 h post-reperfusion for transcriptomic analysis. Total RNA was extracted, sequenced, and aligned to the reference genome. DEGs were identified using the criteria of |fold change| > 2 and FDR < 0.05. To explore the biological functions and regulatory pathways influenced by SM-CDs, KEGG pathway enrichment analysis, GO enrichment analysis, and GSEA were performed on the DEGs.

To further elucidate the potential mechanism by which *Salvia miltiorrhiza*-derived CDs inherit bioactivity from their precursor, network pharmacology analysis was conducted. Active components and corresponding target genes of *Salvia miltiorrhiza* were collected from public databases including TCMSP, SwissADME, and SwissTargetPrediction. Myocardial I/R injury-related genes were retrieved from GeneCards, OMIM, and DisGeNET databases. The intersection of herb-related targets and disease-related genes yielded candidate therapeutic targets, which were subsequently subjected to KEGG pathway enrichment. Finally, overlapping pathways between the transcriptomics-derived DEG enrichments and network pharmacology predictions were analyzed to assess consistency between SM-CDs effects and *Salvia miltiorrhiza*'s pharmacological profiles. Detailed sequencing, bioinformatics pipelines, and database search parameters are provided in the Supplementary Information.

## Supplementary Material

Supplementary methods, figures and tables.

## Abbreviations

MI: myocardial infarction
I/R: ischemia-reperfusion
ROS: reactive oxygen species
CD: carbon dot
SOD: superoxide dismutase
•O_2_^-^: superoxide anion radicals
SM: *Salvia miltiorrhiza*
TEM: transmission electron microscopy
AFM: atomic force microscopy
XRD: X-Ray diffraction
XPS: X-ray photoelectron spectroscopy
•OH: hydroxyl radical
EPR: electron paramagnetic resonance
FTIR: Fourier-transform infrared
^1^H-NMR: ^1^H-nuclear magnetic resonance
DCFH-DA: 2',7'-dichlorofluorescin diacetate
DHE: dihydroethidium
LPS: lipopolysaccharide
iNOS: inducible nitric oxide synthase
OGD/R: oxygen-glucose deprivation/reperfusion
ΔΨm: mitochondrial membrane potential
LAD: left anterior descending
TTC: 2,3,5-triphenyltetrazolium chloride
PCA: principal component analysis
KEGG: Kyoto Encyclopedia of Genes and Genomes
GSEA: gene set enrichment analysis
GO: gene ontology
