# Supplementary material for: Pharmacologically inherited carbon dots from Salvia miltiorrhiza with potent antioxidant activity and multi-pathway modulation for myocardial ischemia-reperfusion injury therapy
Source: Theranostics. 2026 Jan 1;16(4):1855–76. doi: 10.7150/thno.123141 (PMC12680534; doi:10.7150/thno.123141)
Supplement: Supplementary file 1 — Supplementary methods, figures and tables. [file thnov16p1855s1.pdf]

Supporting Information

Pharmacologically Inherited Carbon Dots from *Salvia Miltiorrhiza* with Potent Antioxidant Activity and Multi-Pathway Modulation for Myocardial Ischemia–Reperfusion Injury Therapy

*Kai Zhang[1, #], Zhenyuan Wang[1, #], Letong Zhang[1], Hao Wu[1], Jing Liu[2], Mingzhen Zhang[3, *], Zhichao Deng[3, *], Ruina Liu[1,2, *]*

[1] NHC Key Laboratory of Forensic Science, Department of Forensic Pathology, College of Forensic Medicine, Xi'an Jiaotong University, 76 Yanta West Road, Xi'an, 710061, People's Republic of China.

[2] Center for Translational Medicine, Shaanxi Provincial Key Laboratory of Biological Psychiatry, Department of Psychiatry, The First Affiliated Hospital of Xi'an Jiaotong University, 277 Yanta West Road, Xi'an, 710061, People's Republic of China.

[3] School of Basic Medical Sciences, Xi'an Jiaotong University, 76 Yanta West Road, Xi'an, 710061, People's Republic of China.

[#] *The authors contributed equally to this work.*

* Mingzhen Zhang: Email: mzhang21@xjtu.edu.cn

* Zhichao Deng: Email: dengz1786@gmail.com

* Ruina Liu: Email: liuruina@xjtufh.edu.cn

Experimental Section (Detailed version)

Synthesis of SM-CDs

SM-CDs were synthesized via a hydrothermal method using *Salvia miltiorrhiza* as the carbon precursor. Briefly, 2.5 g of pulverized *Salvia miltiorrhiza* roots were homogenized in 50 mL of ultrapure water under stirring. The homogeneous suspension was transferred into a Teflon-lined stainless-steel autoclave and subjected to hydrothermal treatment at 240°C for 12 h. After naturally cooling to room temperature, the crude product was centrifuged, and the supernatant was sequentially filtered through a 0.22 μm polyethersulfone membrane. To remove residual small-molecule impurities, the filtrate was dialyzed against ultrapure water via a 1 kDa molecular weight cutoff (MWCO) dialysis membrane for 72 h, with the water replaced every 12 h. The resulting SM-CDs solution was then lyophilized to obtain the final powdered product.

Characterization of SM-CDs

The morphology of the SM-CDs was characterized by high-resolution transmission electron microscopy (HRTEM, Tecnai G2 F30 S-TWIN, FEI) and atomic force microscopy (AFM). The Raman spectroscopy of SM-CDs was recorded using a Horiba LabRAM HR Evolution. UV–vis absorption spectra were recorded in the range of 200–800 nm using a SPECORD 200 PLUS spectrophotometer (Analytik Jena). The fluorescence properties, including excitation/emission spectra and fluorescence lifetimes, were analyzed using a fluorescence spectrometer (FLS980, Edinburgh Instruments) equipped with time-correlated single-photon counting (TCSPC) capability. The elemental composition and chemical structure were investigated via X-ray photoelectron spectroscopy (XPS, ESCALAB 250Xi, Thermo Scientific). Surface functional groups were further characterized by Fourier-transform infrared spectroscopy (FTIR, Nicolet 6700, Thermo Scientific) using KBr pelletized samples.

Additionally, ^{1}H-nuclear magnetic resonance (NMR) spectra were obtained using a 600 MHz AVANCE III HD spectrometer (Bruker), with deuterium oxide (D_2O) as the solvent for both pristine and chemically modified SM-CDs. The hydrodynamic size, zeta potential, and polydispersity index (PDI) were detected using a nanoparticle size and zeta potential analyzer (Zetasizer Nano ZSE, Malvern Instruments, UK).

ABTS, Hydroxyl, and Superoxide free radical scavenging activity

A commercial total antioxidant capacity assay kit (Beyotime Biotechnology, Cat. S0119), in which 2,2'-azino-bis(3-ethylbenzothiazoline-6-sulfonic acid) (ABTS) serves as the chromogenic agent, was used. The total antioxidant capacity of SM-CDs at concentrations ranging from 5 to 100 μg/mL was quantified by measuring the absorbance of ABTS$\bullet^{+}$ radicals at 405 nm.

The hydroxyl radical (•OH) scavenging activity of SM-CDs was evaluated using two methods. Method 1: A Fenton reaction system containing Fe^{2+} (10 mM), H_2O_2 (100 mM), 3,3',5,5'-tetramethylbenzidine (TMB, 3 mM), and SM-CDs (50–200 μg/mL) was used to generate •OH, which oxidizes TMB to oxTMB. The •OH scavenging efficacy of SM-CDs was determined by monitoring the reduction in oxTMB absorbance at 652 nm. Method 2: •OH generated via the Fenton reaction was trapped by 5,5-dimethyl-1-pyrroline N-oxide (DMPO), forming DMPO/•OH spin adducts. Electron paramagnetic resonance (EPR) spectra were recorded using an EPR spectrometer (EPR200M, CIQTEK Co., Ltd.) to assess radical scavenging.

The $\bullet O_2^-$ scavenging capacity of SM-CDs was assessed using the three methods. Method 1: $\bullet O_2^-$ was generated in a solution containing riboflavin (6.67 μM), L-methionine (4.33 mM), and nitrotetrazolium blue chloride (NBT, 25 μM) in PBS (0.01 M, pH 7.4), with SM-CDs tested at concentrations ranging from 5 to 200 μg/mL. $\bullet O_2^-$ reduces NBT to blue formazan, and the scavenging

effect of SM-CDs was evaluated by measuring the decrease in absorbance at 560 nm. Method 2: Using a commercial SOD Assay Kit-WST (DOJINDO LABORATORIES, Cat. S311), SM-CDs (5–200 μg/mL) were tested for their ability to inhibit $•O_2^-$-mediated reduction of water-soluble tetrazolium salt (WST-1) to formazan, with absorbance measured at 450 nm. Method 3: $•O_2^-$ generated via the riboflavin/light system was trapped by 5-tert-butoxycarbonyl-5-methyl-1-pyrroline N-oxide (BMPO), forming BMPO/$•O_2^-$ spin adducts. The intensity of EPR signals was analyzed (EPR200M, CIQTEK Co., Ltd.) to assess the $•O_2^-$ scavenging capacity of SM-CDs.

Surface modification of SM-CDs

Synthesis of CDs-PS: SM-CDs (5 mg) were dispersed in 1 mL ultrapure water, followed by the addition of 10 mL dioxane, 1 g 1,3-propanesultone (PS), and 1 mL triethylamine. The mixture was vortexed at 40°C for 24 h. The solvents were removed by rotary evaporation, and the residue was redissolved in ultrapure water. The solution was dialyzed against 0.1 M NaCl for 24 h, followed by dialysis against ultrapure water for 72 h (1 kDa MWCO membrane, 3–4 buffer changes daily). The final product, denoted as CDs-PS, was obtained by lyophilization.

Synthesis of CDs-PS-Hy: CDs-PS (5 mg) were dissolved in 10 mL 0.5 M NaOH and stirred at 40°C for 24 h. The pH of the reaction mixture was neutralized with HCl, and the solution was dialyzed against ultrapure water for 72 h (with a 1 kDa MWCO membrane, 3–4 buffer changes daily), and the product was lyophilized to obtain CDs-PS-Hy.

Synthesis of Re-CDs: SM-CDs (20 mg) were treated with 50 mL of 0.5 M $NaBH_4$ under stirring at 25°C for 24 h. The solution was neutralized with HCl, dialyzed against ultrapure water for 72 h (1 kDa MWCO membrane, 3-4 buffer changes daily), and lyophilized to obtain Re-CDs.

Synthesis of Re-CDs-PS-Hy: Re-CDs-PS-Hy were synthesized following the same procedures used

for CDs-PS and CDs-PS-Hy, with Re-CDs as the starting material.

Cell culture

The mouse macrophage cell line RAW264.7 (RRID: CVCL_0493) and rat cardiomyocyte cell line H9C2 (RRID: CVCL_0286) were purchased from the China Center for Type Culture Collection (CCTCC, Wuhan, China). Both cell lines were authenticated by the supplier and tested negative for mycoplasma contamination prior to experimentation. Both cell lines were cultured in high-glucose Dulbecco's modified Eagle's medium (DMEM) supplemented with 10% fetal bovine serum (FBS) and 1% penicillin-streptomycin. The cells were maintained at 37°C in a humidified incubator with 5% CO_2.

Cell viability assay

The cytotoxicity and biocompatibility of SM-CDs toward RAW 264.7 and H9C2 cells were assessed using a commercial Cell Counting Kit-8 (CCK-8, Beyotime, Cat. C0038). Cells were seeded into 96-well plates. After cell adherence, the culture medium was then replaced with fresh medium containing SM-CDs at various concentrations. After 24 h of incubation, cell viability was evaluated following the manufacturer's protocol, and absorbance at 450 nm was measured using a microplate reader.

Cellular uptake

SM-CDs were fluorescently labeled with Cy5.5 via carbodiimide-mediated conjugation. Briefly, 100 μL of 10 mg/mL EDC (1-ethyl-3-(3-dimethylaminopropyl)carbodiimide) and 200 μL of 10 mg/mL NHS (N-hydroxysuccinimide) were mixed in PBS and activated at 25°C for 30 min. Then, 5 mg SM-CDs were dispersed in 1 mL PBS and reacted with the pre-activated EDC/NHS solution under gentle stirring for 1 h at room temperature in the dark. Then, 0.5 mg sulfo-Cyanine5.5 amine

(MedChemExpress, HY-D1376A) dissolved in 2 mL PBS was added, and the reaction mixture was stirred for 24 h in the dark. The resulting solution was dialyzed against ultrapure water for 24 h using a 1 kDa MWCO membrane to remove unreacted dyes, yielding Cy5.5-labeled CDs (Cy5.5-CDs).

For SM-CDs uptake analysis, RAW 264.7 or H9C2 cells were seeded into 12-well plates. After cell adhesion, 20 μL of Cy5.5-CDs solution was added to each well and incubated for 1, 2, 4, or 6 h. The cells were then harvested and analyzed using a NovoCyte flow cytometer (ACEA Biosciences) to quantify intracellular Cy5.5 fluorescence intensity. For subcellular localization analysis, cells incubated with Cy5.5-CDs for 4 h were stained with Mito-Tracker (Beyotime, Cat. C1048), Lyso-Tracker (Beyotime, Cat. C1047S), and DAPI (Beyotime, Cat. C1005) to label mitochondria, lysosomes, and nuclei, respectively. Cells grown on coverslips were fixed and imaged under laser scanning confocal microscope to assess the co-localization of Cy5.5-CDs.

Intracellular ROS scavenging and Antioxidant Activity

RAW 264.7 or H9C2 cells were seeded into 12-well plates. After adhesion, cells were treated with SM-CDs at concentrations of 5 μg/mL, 10 μg/mL, or 20 μg/mL for 4 h. RAW 264.74 cells were then stimulated with 500 μM H_2O_2 for 4 h, while H9C2 cells were subjected to oxygen–glucose deprivation/reperfusion (OGD/R). For the OGD/R model, the cells were incubated in glucose-free DMEM under hypoxic conditions (1% O_2, 5% CO_2) for 6 h, followed by reperfusion with high-glucose DMEM containing 10% FBS in a normoxic incubator for 12 h. After treatment, cells were gently washed three times with PBS and incubated with DMEM containing either 2′,7′-dichlorodihydrofluorescein diacetate (DCFH-DA, Beyotime, Cat. S0035S) or dihydroethidium (DHE, Beyotime, Cat. S0063) fluorescent probes (diluted 1:1000) for 30 min to label the intracellular ROS or $\bullet O_2^-$, respectively. The ROS and $\bullet O_2^-$ scavenging abilities of SM-CDs were evaluated by flow

cytometry and fluorescence microscopy. In addition, lipid peroxidation levels were assessed using a commercial kit (Beyotime, Cat. S0131S) by quantifying malondialdehyde (MDA), a terminal product of lipid peroxidation. Following the treatments, cells were harvested and lysed, and MDA levels were measured at 532 nm according to the manufacturer's instructions. Absolute concentrations were calculated based on a standard MDA curve. The total protein content was determined using a BCA protein assay kit (Beyotime, Cat. P0010S). The final MDA values were normalized to cellular protein content and expressed as μmol/g protein.

Intracellular anti-inflammatory effect

The anti-inflammatory effects of SM-CDs were evaluated in RAW 264.7 macrophages. The cells were seeded into 12-well plates and pretreated with 5 or 10 μg/mL SM-CDs for 4 h, followed by stimulation with 500 ng/mL lipopolysaccharide (LPS) for 12 h to induce an inflammatory response. Untreated RAW 264.7 cells (without LPS stimulation) served as negative controls. Anti-inflammatory effect was assessed via flow cytometry, RT–qPCR, and Western blotting.

For flow cytometry analysis, cells were collected and incubated with anti-mouse CD16/32 antibody (0.5 mg/mL, BioLegend, Cat. 101301) on ice for 10 min to block Fc receptors. Subsequently, the cells were stained on ice for 30 min with FITC-conjugated anti-mouse CD11b (0.5 mg/mL, BioLegend, Cat. 101205) and APC-conjugated anti-mouse CD86 (0.2 mg/mL, BioLegend, Cat. 105012) to label surface CD11b (macrophage marker) and CD86 (M1 polarization marker), respectively. After centrifugation, the cells were resuspended in PBS, and the proportion of $CD11b^{+}$ $CD86^{+}$ cells (indicating M1 pro-inflammatory macrophages) was analyzed using flow cytometry.

For gene expression analysis, total RNA was extracted using a commercial RNA isolation kit (Beyotime, Cat. R0027), and reverse transcription was performed using a cDNA synthesis kit

(ABclonal, RK20408). Quantitative real-time PCR (ABclonal, RK21203) was then performed to quantify mRNA expression of M1 macrophage markers (iNOS) and pro-inflammatory cytokines (IL-6, IL-1β, TNF-α). Primer sequences are listed in Table S1.

For Western blot analysis, the cells were lysed in RIPA buffer (Beyotime, Cat. P0013B) supplemented with protease and phosphatase inhibitors (Beyotime, Cat. P1046). Total protein concentrations were quantified using a BCA assay kit (Beyotime, Cat. P0010S). Equal amounts of protein (20–30 μg per lane) were separated by 10% SDS-PAGE and transferred onto Polyvinylidene fluoride (PVDF) membranes. Membranes were blocked with 5% non-fat milk in TBST (Tris-buffered saline with 0.1% Tween-20) for 1 h at room temperature and then incubated overnight at 4°C with primary antibodies diluted in the blocking buffer. After washing with TBST, membranes were incubated with HRP-conjugated secondary antibodies for 1 h at room temperature. Protein bands were visualized using an enhanced chemiluminescence (ECL) detection system. Band intensities were quantified using ImageJ software, with β-tubulin serving as the internal loading control. The following primary antibodies were used: iNOS (1:2000, Proteintech, Cat. 18985-1-AP), Beta tubulin (1:5000, Proteintech, Cat. 10094-1-AP).

Intracellular anti-apoptotic effect

The anti-apoptotic effects of SM-CDs were evaluated in H9C2 cells subjected to the OGD/R procedure mentioned above. H9C2 cells were seeded into 12-well plates and pretreated with 10 or 20 μg/mL SM-CDs for 4 h, followed by the OGD/R treatment. The cells without OGD/R treatment served as negative controls.

The mitochondrial membrane potential (ΔΨm) was measured using a JC-1 kit (Beyotime, Cat. C2006). Briefly, the cells were incubated with 500 μL JC-1 staining solution at 37°C for 20 min,

washed twice with JC-1 buffer, and imaged under a fluorescence microscope. JC-1 monomers (green fluorescence) and aggregates (red fluorescence) were quantified using ImageJ software. The $\Delta\Psi m$ loss was expressed as the ratio of green fluorescence to total fluorescence [green / (green + red)]. The ATP production level was also determined using a commercial ATP assay Kit（Beyotime, Cat. S0026）to evaluate mitochondrial function.

For apoptosis detection, Annexin V-FITC/PI staining was performed using a commercial kit (Beyotime, Cat. C1062). Both adherent and detached cells (to include apoptotic/necrotic populations) were collected, stained with Annexin V-FITC and propidium iodide (PI) for 20 min at room temperature in the dark, and analyzed by flow cytometry. Late apoptotic cells were defined as Annexin V^{+}/PI^{+} populations.

Besides, DNA fragmentation was assessed using a TUNEL assay kit (Beyotime, Cat. C1089). Cells were fixed with 4% paraformaldehyde for 30 min, permeabilized with 0.3% Triton X-100 for 5 min, and incubated with TUNEL reaction mixture at 37°C for 60 min in the dark. Subsequently, the cells were stained with FITC-phalloidin (100 nM, 37°C, 60 min, dark, Solarbio, Cat. CA1620). After washing with PBS, the cell nuclei were counterstained with DAPI. TUNEL-positive apoptotic cells were counted under a fluorescence microscope.

For Western blot analysis, the protein levels of cleaved caspase-3, Bax (pro-apoptotic), and Bcl-2 (anti-apoptotic) were evaluated as previously described. The following primary antibodies were used: cleaved caspase-3 (1:1000, Proteintech, Cat. 25128-1-AP), Bax (1:2000, Proteintech, Cat. 50599-2-Ig), Bcl-2 (1:1000, Proteintech, Cat. 26593-1-AP), and β-actin (1:20,000, Proteintech, Cat. 66009-1-Ig).

Animals

All experimental procedures involving animals were conducted following the ethical guidelines approved by the Animal Ethics Committee of Xi'an Jiaotong University and complied with the protocols of the Institutional Animal Care and Use Committee (IACUC) of the university (No: XJTUAE2024-785). Male Sprague–Dawley (SD) rats (body weight: 250 ± 20 g) were obtained from the Experimental Animal Center of Xi'an Jiaotong University. The animals were housed under standardized conditions, including a 12-hour light/dark cycle, ambient temperature of 20–25°C, and relative humidity of 40–70%. Food and water were provided ad libitum. To eliminate potential confounding effects from estrogen fluctuations, only male rats were used in this study.

Myocardial I/R injury model

Briefly, the rats were anesthetized with 2% isoflurane via inhalation. Under aseptic conditions, a left thoracotomy was performed to expose the heart, and the left anterior descending coronary artery (LAD) was ligated with a 7–0 silk suture for 60 min to induce ischemia. Reperfusion was initiated by gently removing the suture to restore blood flow. Meanwhile, SM-CDs (2 mg/mL, 5 μL or 10 μL) were immediately injected into the ischemic anterior wall of the left ventricle with a 32 G needle at the onset of reperfusion. Subsequently, the chest was surgically closed. Throughout the surgical procedure, body temperature was maintained at 37°C using a heating pad. Sham-operated rats underwent identical surgical steps except for LAD ligation. Postoperative analgesia was provided by subcutaneous administration of buprenorphine (0.05 mg/kg), and animals were monitored for 48 hours.

Myocardial enzyme spectrum

Blood samples were collected into anticoagulant-free tubes. After allowing clot formation at room temperature for 30 minutes, samples were centrifuged at 3000 rpm for 5 minutes at 4°C to separate the serum. Serum concentrations of cardiac injury biomarkers—creatine kinase (CK), creatine kinase-MB

isoenzyme (CK-MB), and lactate dehydrogenase-1 isoenzyme (LDH-1)—were measured using an automated biochemical analyzer (Rayto, Chemray 240).

***In vivo* mitochondrial function**

The ΔΨm and ATP production levels in cardiac tissue were examined to evaluate mitochondrial function *in vivo*. For ΔΨm detection, cardiac tissue was immediately excised after sacrifice, and mitochondria were isolated and purified using a commercial tissue mitochondria isolation kit (Beyotime, Cat. C3606). The purified mitochondria were then incubated with the staining working solution of the JC-1 mitochondrial membrane potential assay kit (Beyotime, Cat. C2006) according to the manufacturer's protocol. Fluorescence intensities of JC-1 monomers and aggregates were measured at 530 nm and 590 nm, respectively, using a fluorescence microplate reader. The ratio of JC-1 aggregates to JC-1 monomers was calculated to represent the mitochondrial membrane potential.

For ATP detection, cardiac tissue was homogenized in lysis buffer at a ratio of approximately 100 µL per 20 mg of tissue. The homogenate was centrifuged at 12,000 × g for 5 min at 4°C, and the supernatant was collected for analysis. ATP levels were measured using a commercial ATP assay kit (Beyotime, Cat. S0026) according to the manufacturer's instructions. A standard curve was established for ATP quantification. Protein concentrations were also determined using a BCA protein assay kit (Beyotime, Cat. P0012). Final ATP levels were normalized to protein content and expressed as µmol/g.

***In vivo* anti-apoptotic effect**

Myocardial tissues were collected and cryosectioned into frozen tissue slices. For apoptosis and cytoskeletal staining, sections were fixed in 4% paraformaldehyde for 40 min, permeabilized with 0.5% Triton X-100 for 5 min, and sequentially incubated with a TUNEL reaction mixture (Beyotime, Cat. C1089; 37°C, 60 min, dark) and FITC-phalloidin (Solarbio, Cat. CA1620; 100 nM, 37°C, 60 min,

dark). After three washes with PBS, cell nuclei were counterstained with DAPI (Beyotime, Cat. C1005). TUNEL-positive apoptotic cells were quantified under a fluorescence microscope. The protein levels of cleaved caspase-3, Bax, and Bcl-2 were analyzed by Western blot following the protocol previously described. Primary antibodies included: cleaved caspase-3 (1:1000, Proteintech, Cat. 25128-1-AP), Bax (1:2000, Proteintech, Cat. 50599-2-Ig), Bcl-2 (1:1000, Proteintech, Cat. 26593-1-AP), and β-actin (1:20,000, Proteintech, Cat. 66009-1-Ig).

TTC-Evans blue staining

The LAD coronary artery was re-occluded at the original ligation site using a 7–0 suture. A vascular clip was then used to clamp the ascending aorta, and retrograde perfusion was performed via the aortic root with 2% Evans Blue dye (Coolaber, Cat. SL7203) to label the perfused (non-ischemic) regions. Hearts were excised, snap-frozen at −20°C for 1 h, and sectioned transversely into 1-mm-thick slice. The tissue slices were incubated in 1% 2,3,5-triphenyltetrazolium chloride (TTC, Coolaber, Cat. SL7141) dissolved in PBS (pH 7.4) for 15 min at room temperature in the dark. After fixation with 4% paraformaldehyde for 30 min, the sections were imaged under a stereomicroscope. ImageJ software was employed to quantify the areas of blue-stained tissue (non-ischemic region), red-stained tissue (ischemic but non-infarct region), and pale tissue (infarct region).

Immunofluorescent staining

Immunofluorescence staining was performed to evaluate M1 macrophage polarization on day 3 and neovascularization in granulation tissue on day 7 after reperfusion. Briefly, the hearts were collected, embedded in OCT compound, and sectioned into frozen slices. After fixation, permeabilization, and blocking, the sections were incubated overnight at 4°C with primary antibodies, followed by incubation with fluorescence-conjugated secondary antibodies at room temperature. The tyramide

signal amplification technique was used for co-labeling CD68 and CD86). Nuclei were counterstained with DAPI, and images were captured using a fluorescence microscope. The proportion of $CD86^+$ cells in $CD68^+$ macrophages and the $CD31^+$ microvessel density in granulation tissue regions were calculated with ImageJ software. The primary antibodies used were as follows: CD86 (1:2000, Proteintech, Cat. 13395-1-AP), CD68 (1:2000, Proteintech, Cat. 28058-1-AP), and CD31 (1:500, Proteintech, Cat. 11265-1-AP).

Echocardiography

Cardiac function was assessed in isoflurane-anesthetized rats using a small animal photoacoustic-ultrasound imaging system (Fujifilm VisualSonics Vevo® LAZR-X). A double-blind protocol was employed where both the operator performing image acquisition and the analyst calculating parameters were blinded to experimental groupings. The left ventricular ejection fraction (LVEF) and left ventricular fractional shortening (LVFS) were calculated using the Vevo LAB software provided by the manufacturer.

Masson's trichrome and picrosirius red staining

Rat hearts were harvested and perfused with PBS to remove residual blood, then cryosectioned into frozen tissue slices. The sections were immersed in 70% ethanol for 2 min. Masson’s trichrome and picrosirius red staining was performed using commercial kits (Beyotime, Cat. C0189S, Cat. C0190S) according to the manufacturer’s protocol. After staining, images of tissue sections were acquired under a light microscope. ImageJ software was used to quantify the thickness and circumferential degree of the fibrotic tissues.

Transcriptomic analysis

Total RNA was extracted from cardiac tissues using the TRIzol method, dissolved in DEPC-treated water, and quantified using a Qubit fluorometer and Qsep400 fragment analyzer. mRNA libraries were constructed through poly(A) selection using Oligo(dT) beads, followed by fragmentation and strand-specific cDNA synthesis incorporating dUTPs. Libraries with insert sizes ranging from 250 to 350 bp were subjected to PCR amplification and quality control, then sequenced on an Illumina platform to generate 150 bp paired-end reads using fluorescence-labeled dNTPs.

For bioinformatic analysis, raw sequencing reads were preprocessed through Fastp for adapter removal and quality filtering (reads with >10% N content or >50% bases with $Q \leq 20$ were discarded). Clean reads were aligned to the reference genome using HISAT2, and gene expression levels were quantified with featureCounts and normalized as fragments per kilobase of transcript per million mapped reads (FPKM).

Differential expression analysis was conducted using DESeq2, with significance determined based on Benjamini–Hochberg-adjusted p-values (false discovery rate, FDR < 0.05). Functional enrichment analyses were performed using both the Kyoto Encyclopedia of Genes and Genomes (KEGG) and Gene Ontology (GO) databases. Enriched KEGG pathways and GO terms were identified through hypergeometric testing, with KEGG evaluated at the pathway level and GO assessed at the term level. Gene Set Enrichment Analysis (GSEA) was performed using the clusterProfiler R package to identify coordinated expression changes across predefined gene sets. Significantly enriched pathways were identified based on the following thresholds: absolute normalized enrichment score ($|NES|$) > 1, p-value < 0.05, and FDR < 0.25.

Network pharmacology analysis

The active constituents of *Salvia miltiorrhiza* were initially screened from the Traditional Chinese

Medicine Systems Pharmacology (TCMSP) database based on criteria of oral bioavailability (OB) ≥30% and drug-likeness (DL) ≥0.18. The canonical SMILES structures of the selected compounds were obtained from the PubChem database and further evaluated using SwissADME to assess pharmacokinetic properties. Only compounds exhibiting high gastrointestinal absorption and fulfilling at least two out of five drug-likeness rules were retained for subsequent analysis. Potential biological targets of the filtered bioactive compounds were predicted using SwissTargetPrediction under default settings. Simultaneously, myocardial I/R injury-related genes were systematically collected from the GeneCards, OMIM, and DisGeNET databases using the keyword "myocardial ischemia reperfusion injury". Overlapping genes between the predicted compound targets and myocardial I/R injury-related genes were identified using the Venny diagram tool. These shared targets were then subjected to KEGG pathway enrichment analysis.

***In vivo* biosafety assessment**

Under anesthesia, the rats underwent thoracotomy to expose the heart. SM-CDs (4 mg/mL, 10 μL, equivalent to twice the dose used in the high-dose treatment groups) were injected into the anterior wall of the left ventricle with a 32 G needle. The control rats received an equivalent volume of sterile saline at the same site. Following surgery, animals were monitored for 14 consecutive days, with daily body weight measurements recorded. On day 1 post-injection, serum samples were collected for evaluation of myocardial injury biomarkers, including CK, CK-MB, and LDH-1. On day 7 and 14, serum levels of liver function markers (ALT, AST) and renal function parameters (BUN, CREA) were quantified using automated biochemical analyzers. Major organs (heart, liver, spleen, lungs, kidneys) were harvested on day 7 or 14, fixed in 4% paraformaldehyde, embedded in paraffin, and sectioned for histological examination. The tissue sections were stained with hematoxylin–eosin (HE) and

examined under a light microscope to assess potential histopathological changes.

Statistical analysis

The quantitative data are presented as mean ± standard deviation (SD). Normality and homogeneity of variance were assessed using Shapiro-Wilk and Levene's tests, respectively. For two-group comparisons, two-tailed Student's t-tests were employed. For comparisons involving multiple groups, one-way analysis of variance (ANOVA) was used, followed by Bonferroni's post hoc test. A p-value of <0.05 was considered statistically significant. All statistical analyses were conducted using SPSS 22.0 (SPSS Inc., USA), and GraphPad Prism 9.5 (GraphPad Software Inc., USA) was used for data visualization.

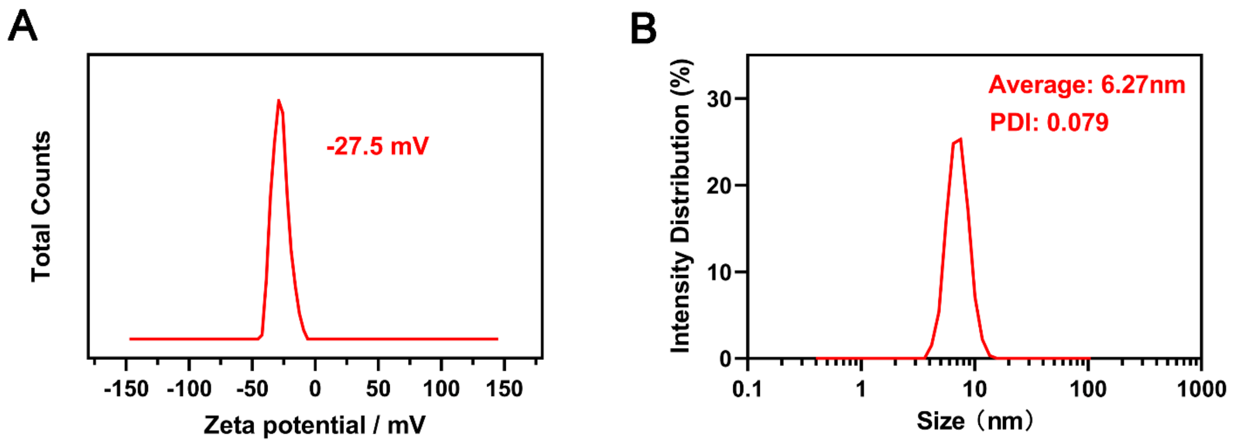

Figure S1. (A) Zeta potential measurement of SM-CDs. (B) Hydrodynamic size and polydispersity index (PDI) of SM-CDs.

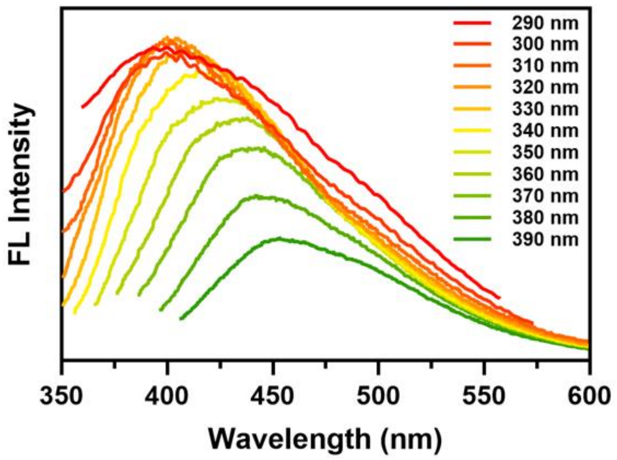

Figure S2. Fluorescence emission spectra of SM-CDs at different excitation wavelengths, demonstrating excitation-dependent emission behavior.

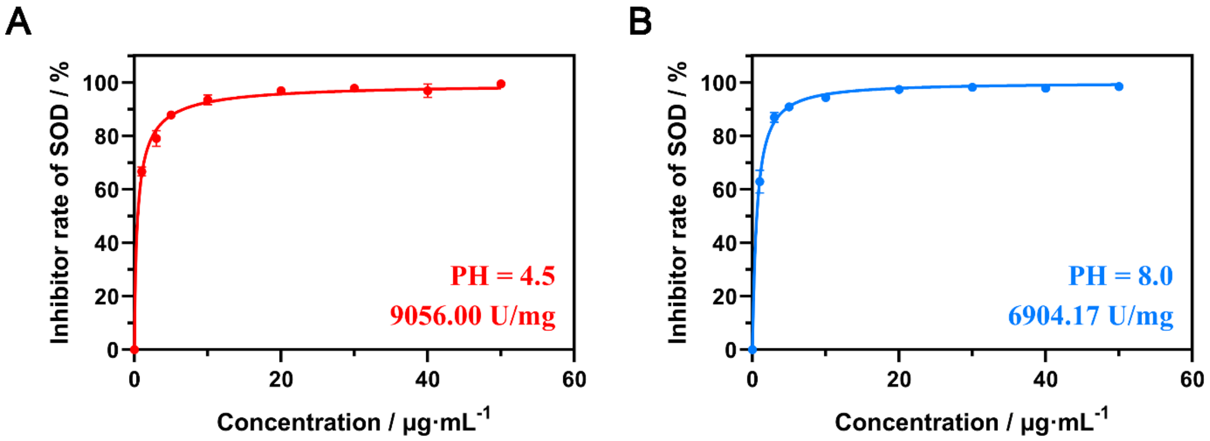

Figure S3. SOD-like activity of SM-CDs under acidic (a) and alkaline (b) conditions.

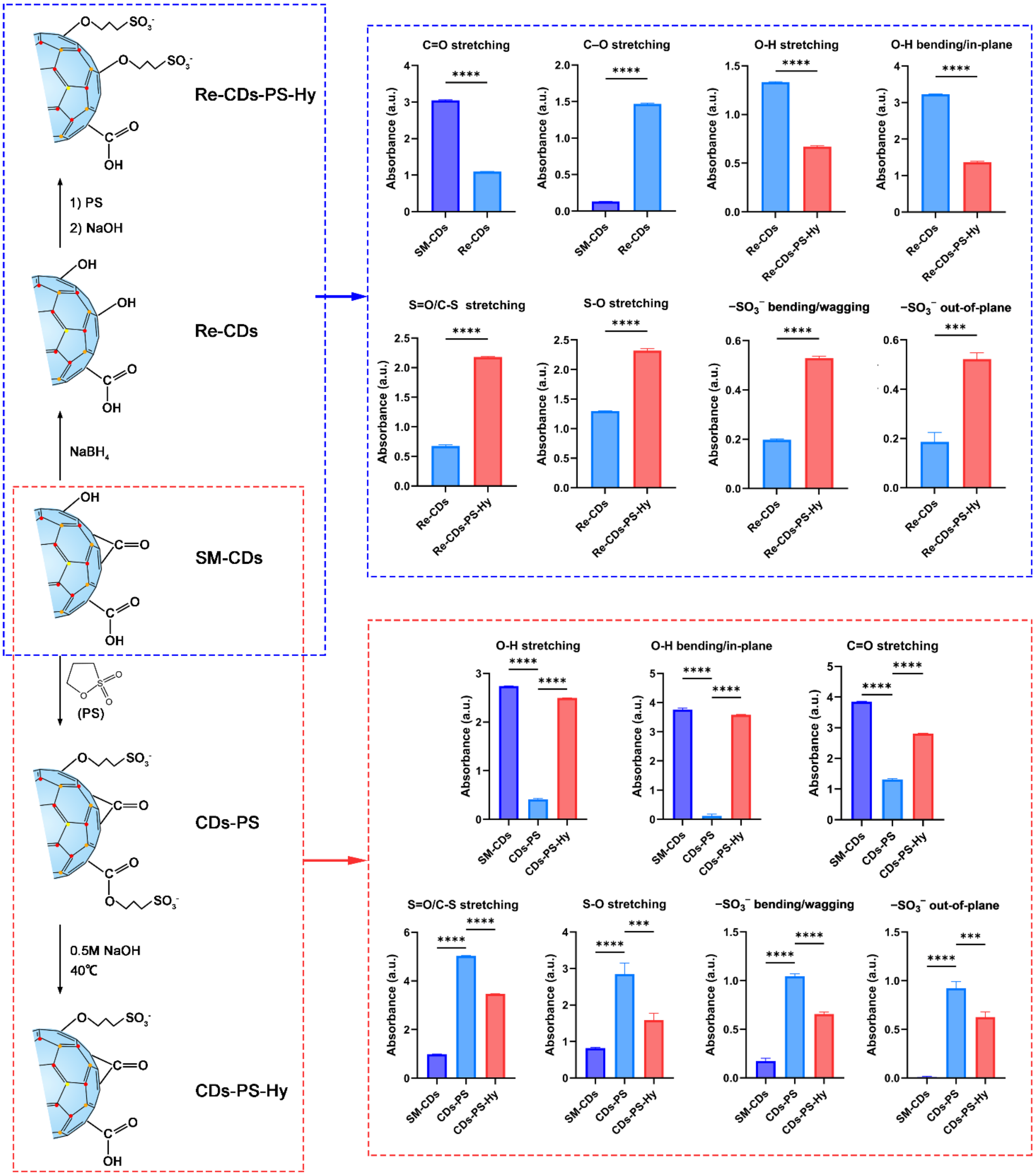

Figure S4. FTIR semi-quantitative analysis of functional group changes during surface modification. Data are analyzed using two-tailed unpaired Student *t*-test and represented as mean ± SEM (n=3 for biologically independent samples). ***: $P < 0.001$. ****: $P < 0.0001$.

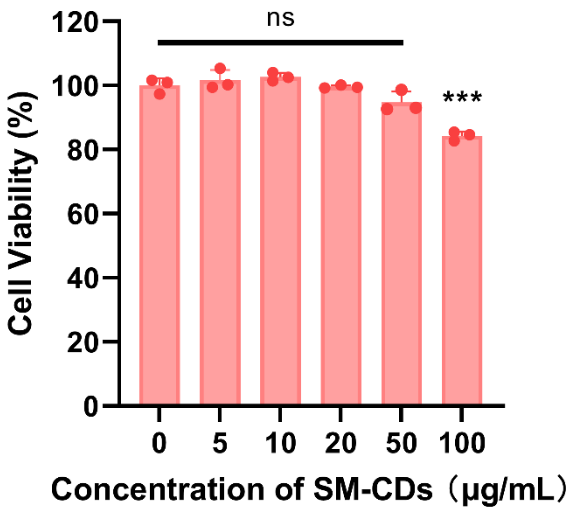

Figure S5. Cytotoxicity evaluation of SM-CDs in RAW264.7 cells. Cell viability was assessed using the CCK-8 assay after 24 h of treatment with various concentrations of SM-CDs. Data are analyzed using One-way ANOVA with Bonferroni post hoc test and presented as mean ± SEM (n = 3 for biologically independent samples). ***: $P < 0.001$. ns: not significant.

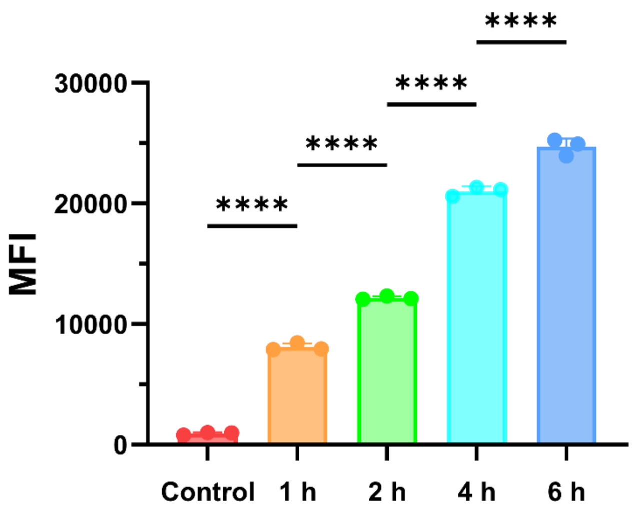

Figure S6. Quantitative comparison of mean fluorescence intensity of Cy5.5-labeled SM-CDs in RAW264.7 cells after co-incubation, indicating their cellular uptake efficiency. Data are analyzed using One-way ANOVA with Bonferroni post hoc test and presented as mean ± SEM (n = 3 for biologically independent samples). ****: $P < 0.0001$.

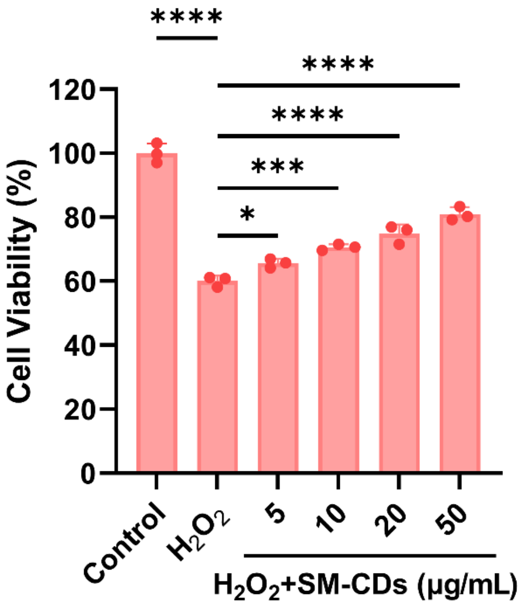

Figure S7. Effect of SM-CDs on RAW264.7 cell viability following H_2O_2 treatment. Cell viability was measured using the CCK-8 assay under various SM-CDs concentrations. Data are analyzed using One-way ANOVA with Bonferroni post hoc test and presented as mean ± SEM (n = 3 for biologically independent samples). *: $P < 0.05$. ***: $P < 0.001$. ****: $P < 0.0001$. ns: not significant.

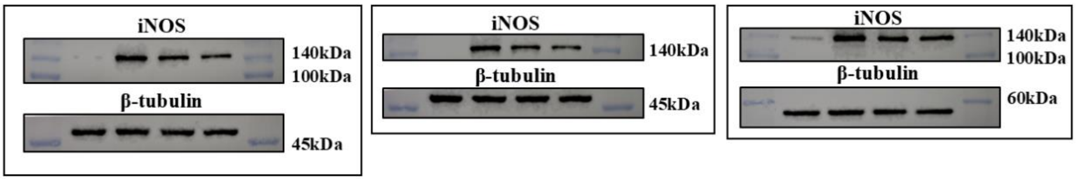

Figure S8. Original western blot images of iNOS expression in RAW264.7 cells.

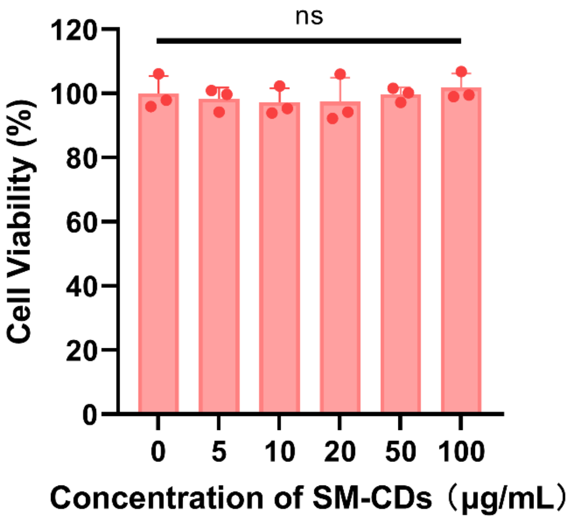

Figure S9. Cytotoxicity evaluation of SM-CDs in cardiomyocytes (H9C2 cells). Cell viability was assessed using the CCK-8 assay after 24 h of treatment with various concentrations of SM-CDs. Data are analyzed using One-way ANOVA with Bonferroni post hoc test and presented as mean ± SEM (n = 3 for biologically independent samples). ns: not significant.

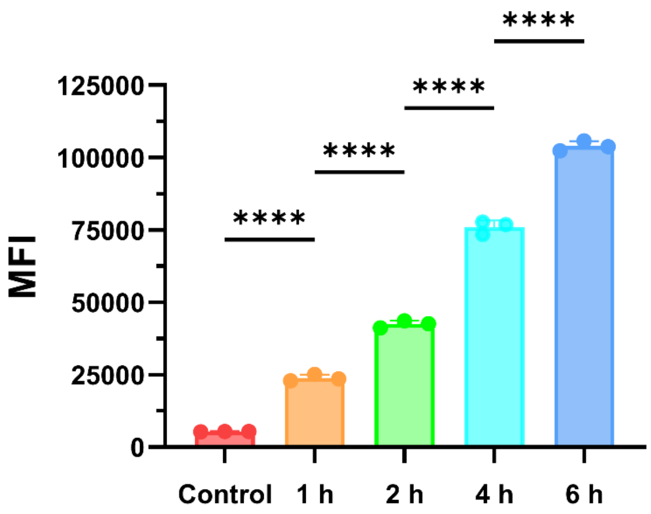

Figure S10. Quantitative comparison of mean fluorescence intensity of Cy5.5-labeled SM-CDs in H9C2 cells after co-incubation, indicating their cellular uptake efficiency. Data are analyzed using One-way ANOVA with Bonferroni post hoc test and presented as mean ± SEM (n = 3 for biologically independent samples). ****: $P < 0.0001$.

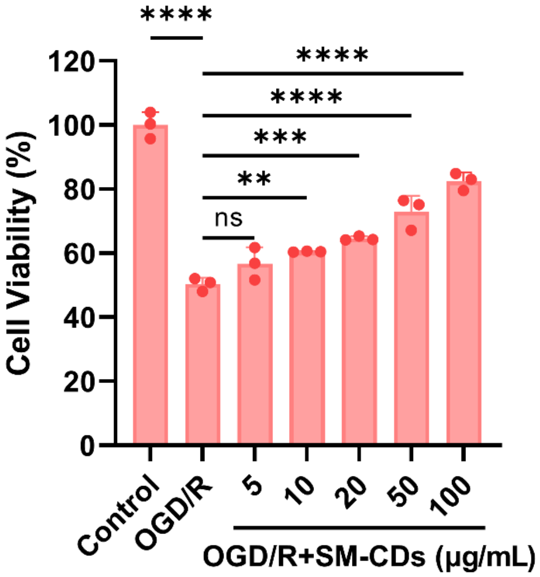

Figure S11. Effect of SM-CDs on H9C2 cell viability following OGD/R injury. Cell viability was measured using the CCK-8 assay under various SM-CDs concentrations. Data are analyzed using One-way ANOVA with Bonferroni post hoc test and presented as mean ± SEM (n = 3 for biologically independent samples). **: $P < 0.01$. ***: $P < 0.001$. ****: $P < 0.0001$. ns: not significant.

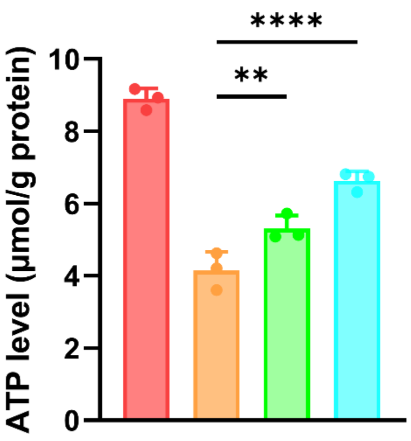

Figure S12. ATP production levels in H9C2 cells following different treatments. Data are analyzed using One-way ANOVA with Bonferroni post hoc test and presented as mean ± SEM (n = 3 for biologically independent samples). **: $P < 0.01$. ****: $P < 0.0001$.

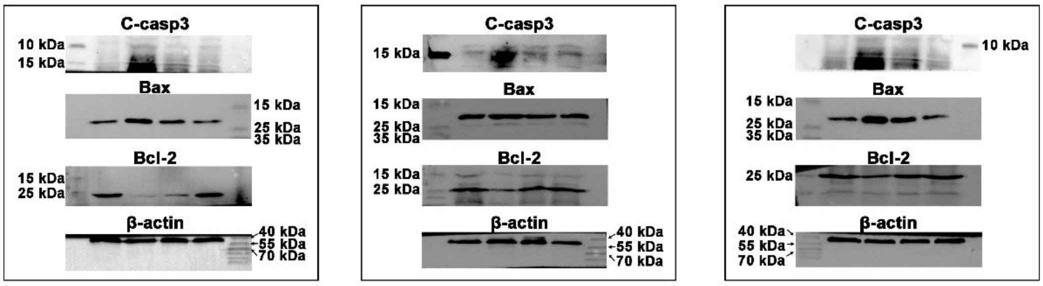

Figure S13. Original Western blot images of cleaved caspase-3, Bax, and Bcl-2 in H9C2 cells.

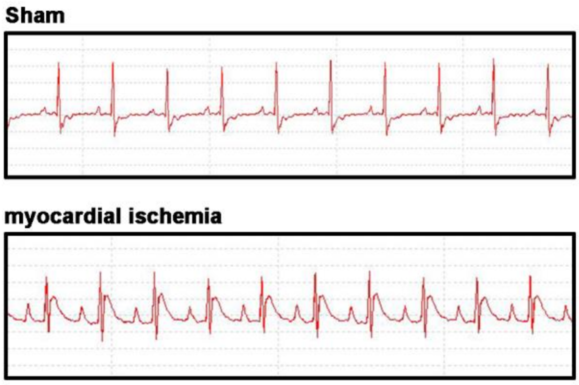

Figure S14. Representative electrocardiogram (ECG) recordings confirming myocardial ischemia during LAD ligation.

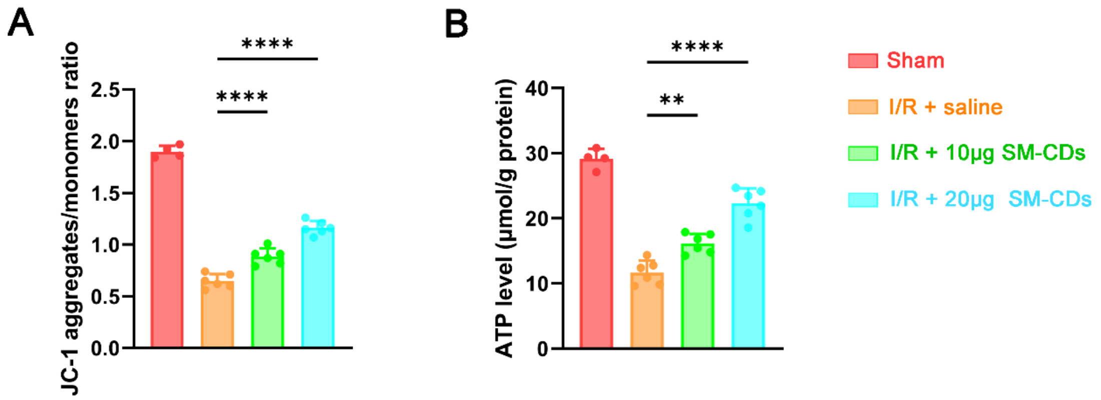

Figure S15. (A) Mitochondrial membrane potential of purified mitochondria, represented as the ratio of JC-1 aggregates to JC-1 monomers, in myocardial tissue after different treatments. (B) ATP production levels in myocardial tissue after different treatments. Data are analyzed using One-way ANOVA with Bonferroni post hoc test and presented as mean ± SEM (n = 4 biologically independent animals for Sham groups and 6 for I/R groups). **: $P < 0.01$. ****: $P < 0.0001$.

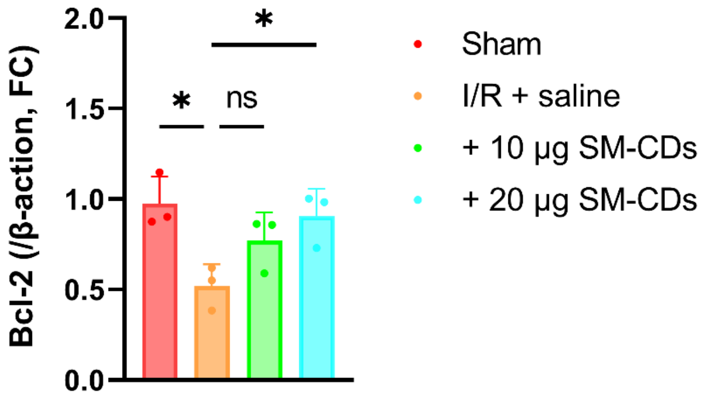

Figure S16. Quantification of Bcl-2 expression by Western blot in myocardial tissue after different treatments. Data are analyzed using One-way ANOVA with Bonferroni post hoc test and presented as mean ± SEM (n = 3 for biologically independent animals). *: $P < 0.05$. ns: not significant.

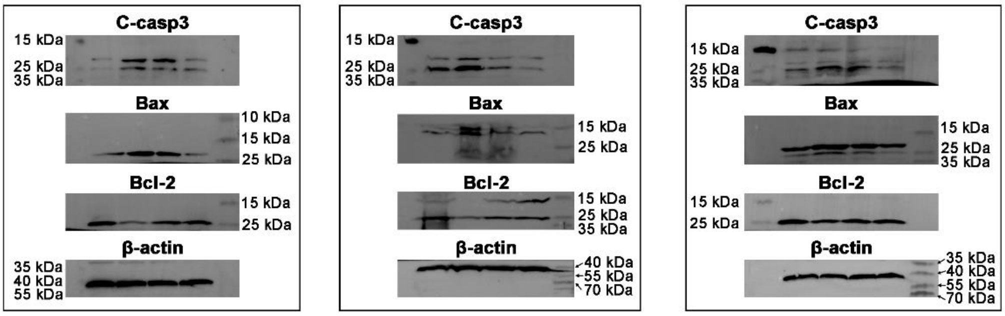

Figure S17. Original Western blot images of cleaved caspase-3, Bax, and Bcl-2 in rat myocardial tissue following I/R injury and SM-CDs treatment.

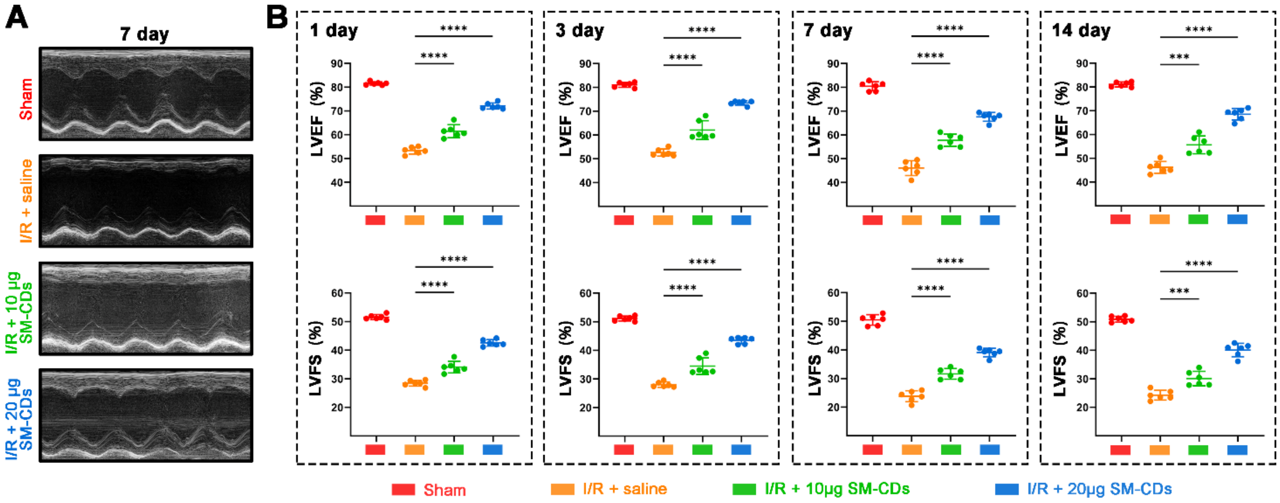

Figure S18. (A) Representative echocardiographic images on days 7 post-reperfusion. (B) Comparison of cardiac function (LVEF and LVFS) in rats at different time points following I/R injury and SM-CDs treatment. Data are analyzed using One-way ANOVA with Bonferroni post hoc test and presented as mean ± SEM (n = 6 for biologically independent animals). ***: $P < 0.001$. ****: $P < 0.0001$.

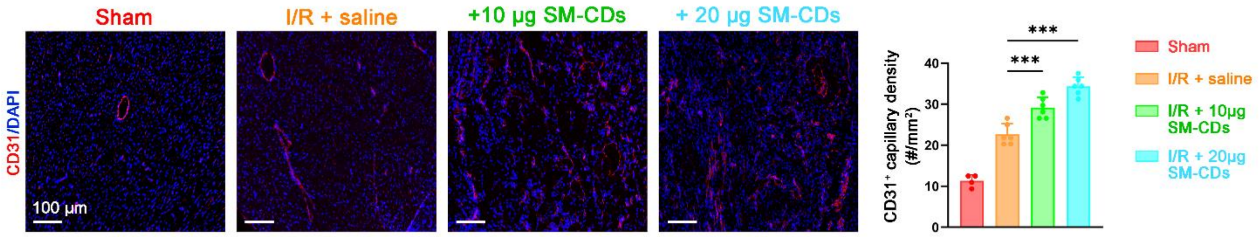

Figure S19. Representative fluorescent images and quantification of $CD31^+$ capillary in the ischemic region on day 7 post-reperfusion. Data are analyzed using One-way ANOVA with Bonferroni post hoc test and presented as mean ± SEM (n = 4 biologically independent animals for Sham groups and 6 for I/R groups). ***: $P < 0.001$.

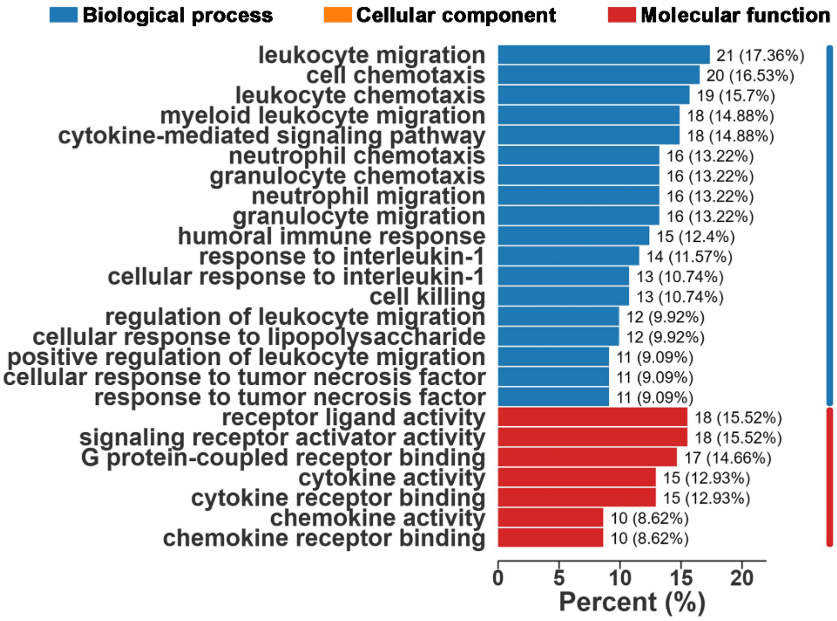

Figure S20. Gene Ontology (GO) enrichment analysis of down-regulated DEGs in the SM-CDs-treated group compared to the untreated I/R group.

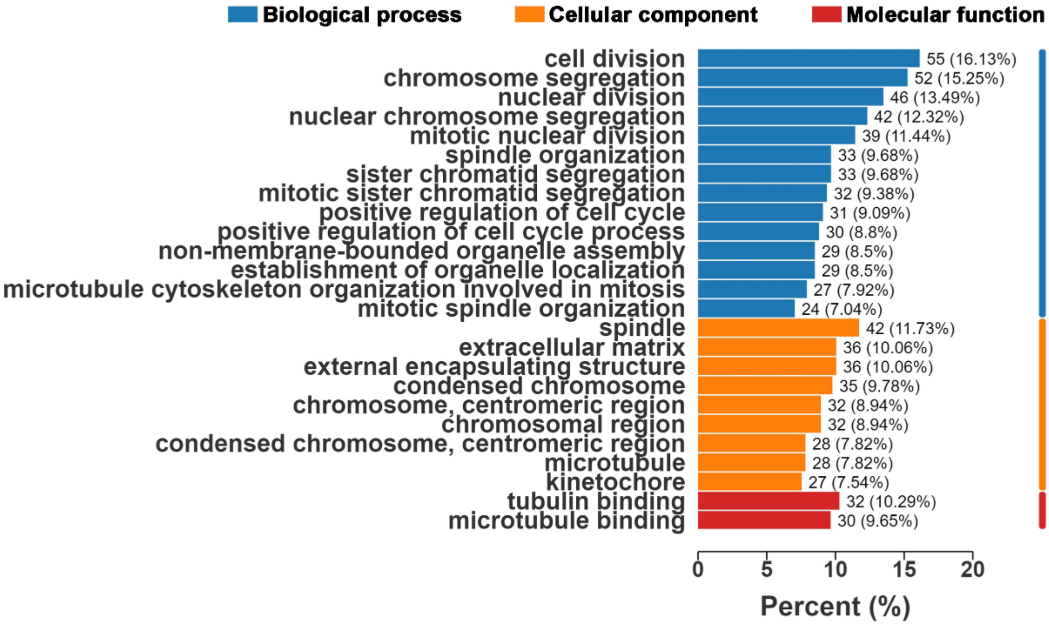

Figure S21. Gene Ontology (GO) enrichment analysis of up-regulated DEGs in the SM-CDs-treated group compared to the untreated I/R group.

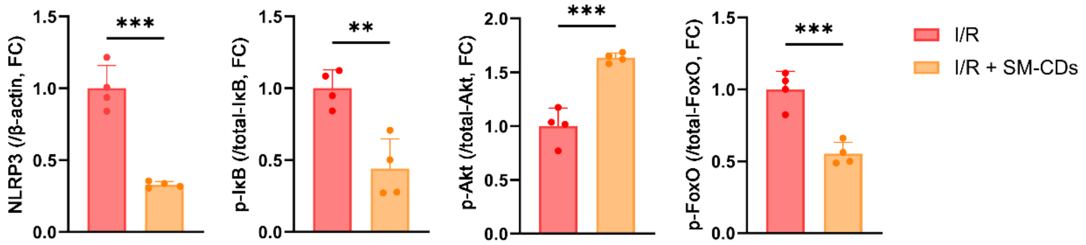

Figure S22. Quantification of Western blot results for NLRP3, p-IκB/total IκB, p-Akt/total Akt, and p-FoxO/total FoxO ratios. Data are analyzed using two-tailed unpaired Student *t*-test and presented as mean ± SEM (n = 4 for biologically independent animals). **: $P < 0.01$. ***: $P < 0.001$.

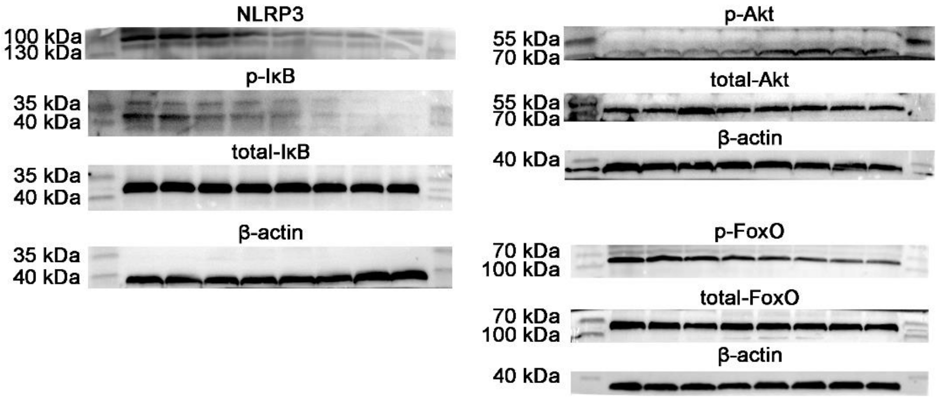

Figure S23. Original Western blot images of NLRP3, p-IκB, total-IκB, p-Akt, total Akt, p-FoxO$_1$, and total FoxO$_1$ in myocardial tissue from I/R rats with or without SM-CDs treatment.

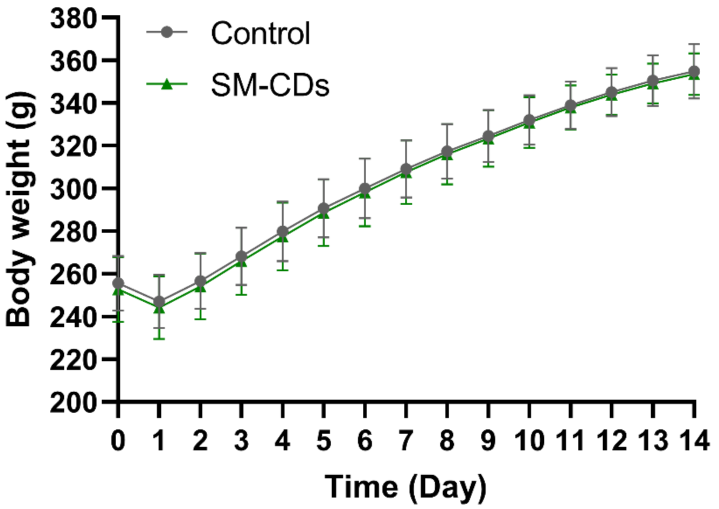

Figure S24. Body weight changes in rats following SM-CDs treatment over a 14-day period compared to untreated controls. Data are analyzed using two-tailed unpaired Student *t*-test and presented as mean ± SEM (n=6 for biologically independent animals). No statistically significant differences were observed.

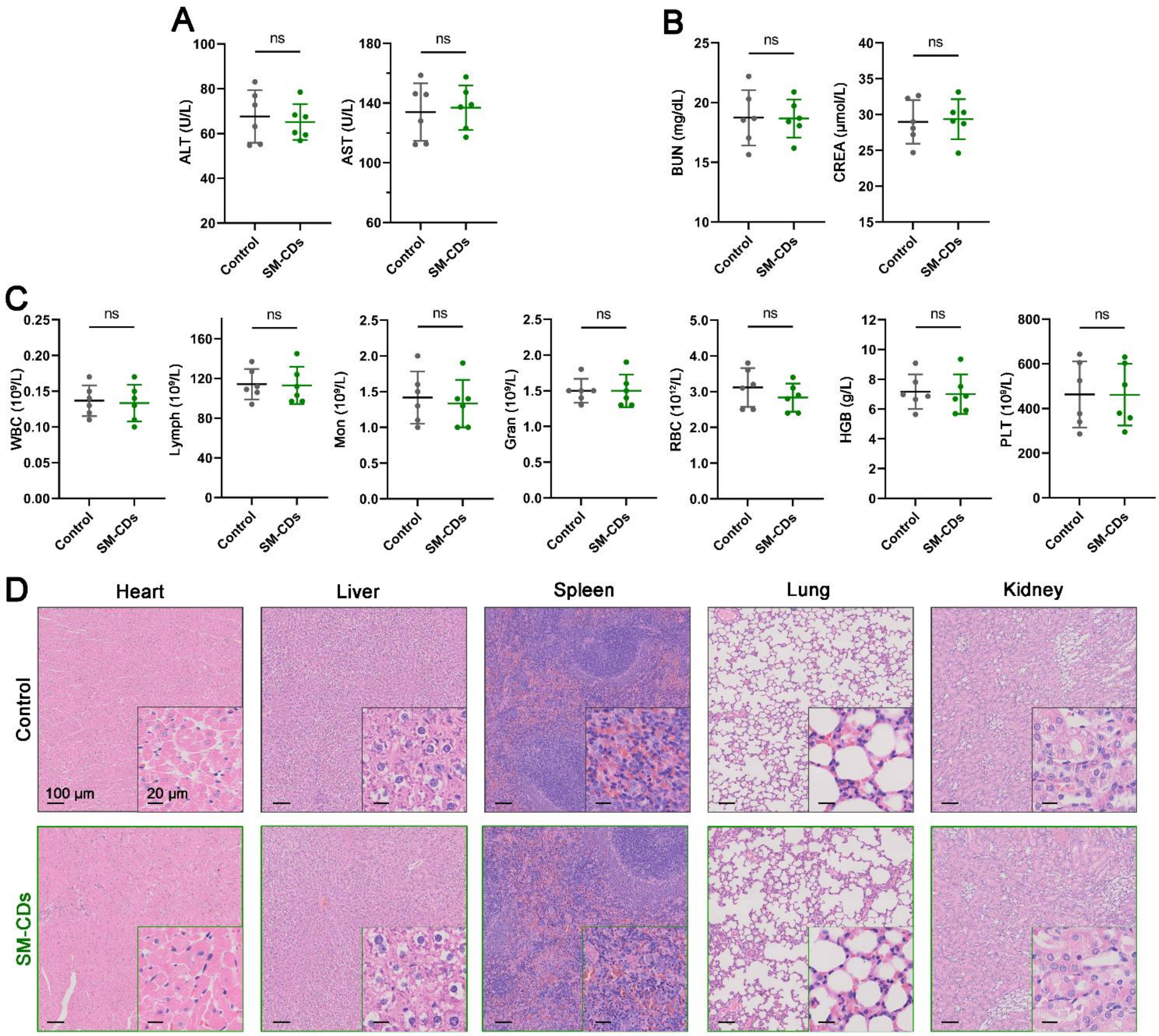

Figure S25. *In vivo* biosafety assessment of SM-CDs on day 7 after SM-CDs administration. (A) Serum levels of liver function indices ALT and AST. (B) Serum levels of renal function indices BUN and CREA. (C) Complete blood count results. (D) Representative H&E-stained images of major organs. Data are analyzed using two-tailed unpaired Student *t*-test and represented as mean ± SEM (n=6 for biologically independent animals). ns: not significant.

Table S1. FTIR band assignments

Band	Assignment
3200 cm^{-1}	O-H stretching vibrations
1600 cm^{-1}	C=O stretching vibrations
1400 cm^{-1}	O-H bending/in-plane vibrations
1200 cm-1	S=O/C-S stretching vibrations
1100 cm^{-1}	C–O stretching vibrations
1060 cm^{-1}	S-O stretching vibrations
610 cm^{-1}	$-SO_3^-$ bending/wagging vibrations
528 cm^{-1}	$-SO_3^-$ out-of-plane vibrations

Table S2. Primer sequences used in this work

Primers	Forward/Reverse	Sequence (5′-3′)
TNF-α	F	GGTGCCTATGTCTCAGCCTCTT
	R	GCCATAGAACTGATGAGAGGGAG
IL-1β	F	TGGACCTTCCAGGATGAGGACA
	R	GTTCATCTCGGAGCCTGTAGTG
IL-6	F	TACCACTTCACAAGTCGGAGGC
	R	CTGCAAGTGCATCATCGTTGTTC
iNOS	F	GAGACAGGGAAGTCTGAAGCAC
	R	CCAGCAGTAGTTGCTCCTCTTC
GAPDH	F	CATCACTGCCACCCAGAAGACTG
	R	ATGCCAGTGAGCTTCCCGTTCAG
HO-1	F	TTTCAGAAGGGTCAGGTGTCC
	R	CTGCTTGTTTCGCTCTATCTCC
NOX-2	F	CCTGTATGTGGCTGTGACTC
	R	TCAAAGTAAGACCTCCGAATGG
β-actin	F	GGCTGTATTCCCCTCCATCG
	R	CCAGTTGGTAACAATGCCATGT
